# Signaling Pathway and Small-Molecule Drug Discovery of FGFR: A Comprehensive Review

**DOI:** 10.3389/fchem.2022.860985

**Published:** 2022-04-14

**Authors:** Jia Zheng, Wei Zhang, Linfeng Li, Yi He, Yue Wei, Yongjun Dang, Shenyou Nie, Zufeng Guo

**Affiliations:** Center for Novel Target and Therapeutic Intervention, Institute of Life Sciences, Chongqing Medical University, Chongqing, China

**Keywords:** FGFR, tyrosine kinase, small-molecule inhibitors, targeted therapy, cancer

## Abstract

Targeted therapy is a groundbreaking innovation for cancer treatment. Among the receptor tyrosine kinases, the fibroblast growth factor receptors (FGFRs) garnered substantial attention as promising therapeutic targets due to their fundamental biological functions and frequently observed abnormality in tumors. In the past 2 decades, several generations of FGFR kinase inhibitors have been developed. This review starts by introducing the biological basis of FGF/FGFR signaling. It then gives a detailed description of different types of small-molecule FGFR inhibitors according to modes of action, followed by a systematic overview of small-molecule-based therapies of different modalities. It ends with our perspectives for the development of novel FGFR inhibitors.

## 1 Introduction

Currently, one of the most important modalities for cancer treatment is the targeted therapy which hampers the growth of cancer cells by chemical intervention against specific target biomolecules known to be essential for tumorigenesis and proliferation. A number of protein kinases in the human body are associated with cancer initiation and progression, and small molecules that inhibit these kinases have thus far gained notable achievement manifested by ∼70 FDA-approved small molecule kinase inhibitor drugs for the treatment of a variety of malignancies (Ayala-Aguilera et al., 2022). FGFRs are a family of receptor tyrosine kinases that have been successfully targeted by three approved small-molecule inhibitors. Due to their functional versatility and frequent alterations in different cancers, FGFRs are considered to be a promising target, and more inhibitors are expected to be translated from bench to bedside in the near future.

Small-molecule FGFR inhibitors have been reviewed by others in the past several years, but these papers mainly focused on small molecules targeting the kinase domain. Herein, we make a systematic and comprehensive description on FGF/FGFR signaling, their role in cancer development, and drug resistance. We also update the development of different modalities targeting FGF-FGFR axis with a detailed discussion of their advantages and future trend.

## 2 Fibroblast Growth Factors

The mammalian fibroblast growth factors (FGFs) are a family of 23 proteins, which exert a wide variety of biological effects on different types of cells. Based on their sequence homology and mode of action, these proteins are classified as secreted FGFs and intracellular FGFs (iFGFs). The iFGFs (FGF11-14) are non-signaling factors that do not bind to any cell surface receptor. Instead, they function as cofactors for voltage-gated sodium channels (Goldfarb et al., 2007). In contrast, all secreted FGFs signal to a class of receptor tyrosine kinases named fibroblast growth factor receptors (FGFRs). In general, secreted FGFs are produced intracellularly and secreted to extracellular matrix (ECM) and eventually bind to FGFRs to initiate signal transduction.

Depending on how far they can travel, secreted FGFs are further classified into two subfamilies: canonical FGFs and endocrine FGFs. In the ECM, canonical FGFs (FGF1-10,16–18, 20, and 22) interact with copious cofactors named heparan sulfate proteoglycans (HSPGs), which limit diffusion of FGFs and regulate their specificity toward FGFRs (Ornitz, 2000; Matsuo et al., 2013). Hence, canonical FGFs function as autocrine or paracrine factors, traveling merely a short distance before binding to the FGFRs on the cells of their origin or adjacent cells (Belov et al., 2013). The binding of canonical FGFs to FGFRs triggers a series of cellular processes related to cellular survival, metabolism, proliferation and differentiation, and consequently mediates organogenesis, tissue metabolism, repair, regeneration and inflammatory response (Belov et al., 2013; Powers et al., 2000; N.; Wang et al., 2018).

Due to the reduced affinity for HSPGs, endocrine FGFs (FGF15/19, 21 and 23) often permeate through the HSPGs-rich extracellular matrix into the circulatory system, and subsequently reach all parts of the body like endocrine hormones (Fernandes-Freitas et al., 2015). Instead of HSPGs, endocrine FGFs require members of Klotho family, including αKlotho, βKlotho, and Klotho-LPH related protein (KLPH), to generate FGF-FGFR-Klotho ternary complex (Angelin et al., 2012; Ding et al., 2012). As a result of their hormone-like features, endocrine FGFs play important roles in the metabolism of bile acid, glucose and lipid in addition to the canonical FGF functions.

Therefore, dysregulation of expression, secretion, and degradation of FGFs lead to aberrations in the metabolism, organogenesis (Dorey et al., 2010; Yu et al., 2017), wound healing (Müller et al., 2012), and are responsible for many cancers (Brooks et al., 2012).

## 3 Fibroblast Growth Factor Receptors

The human fibroblast growth factor receptors belong to receptor tyrosine kinases (RTKs), consisting of FGFR1, FGFR2, FGFR3, FGFR4, and FGFR5. Although FGFR1-4 are encoded by four distinct genes, they share great sequence homology with an identity of 56–71%. The FGFR5, also called FGFR-like 1 (FGFRL1), possesses structural similarity with FGFR1-4 but lacks an intracellular kinase domain (Wiedemann et al., 2000). Activated FGFRs participate in multiple cell processes through intervening several signaling pathways.

### 3.1 Structure of FGFR

**FIGURE 1:**
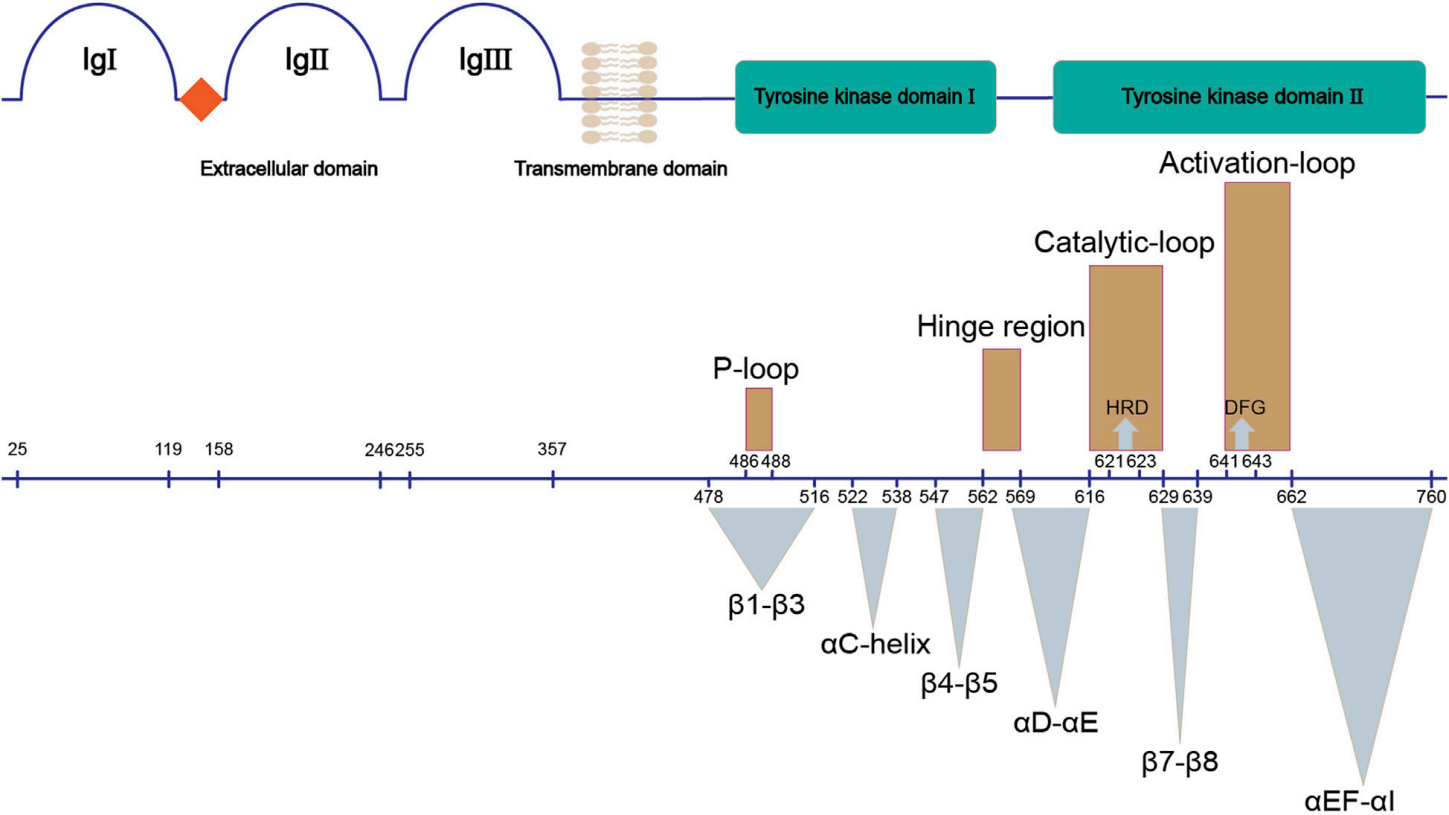
Structure of FGFR. The upper panel shows the main domains of FGFR; the bottom panel demonstrates their corresponding sequences (UniProtKB: P11362). Three dimensional structures (brown) and functional regions (cyan) involved in the kinase domain of FGFR are marked.

FGFRs are single-pass transmembrane proteins containing approximately 800 amino acids, which are composed of several domains: an extracellular ligand binding domain, a transmembrane domain, and an intracellular domain with kinase activity (Figure 1).

The extracellular ligand binding domain of FGFRs is composed of three immunoglobulin-like subdomains (IgI, IgII and IgIII) and an acidic-residues-rich sequence termed acid box (Itoh et al., 2004). IgI and acid box have been demonstrated to play a key role in autoinhibition of FGFRs in the absence of FGFs (Sanchez-Heras et al., 2006). IgII and IgIII form the FGF-binding pocket, thus are responsible for the binding specificity between FGFRs and FGFs. There are two isoforms of IgIII (b/c) in FGFR1-3 that result from alternative splicing, while this is not observed for FGFR4.

The single-pass transmembrane domain (TM) is embedded in the cell membrane, functioning as an anchor of FGFR. The TM also supports the dimerization of cytoplasmic kinase domains of two FGFRs which leads to activation of FGFR (Itoh et al., 2004).

**FIGURE 2:**
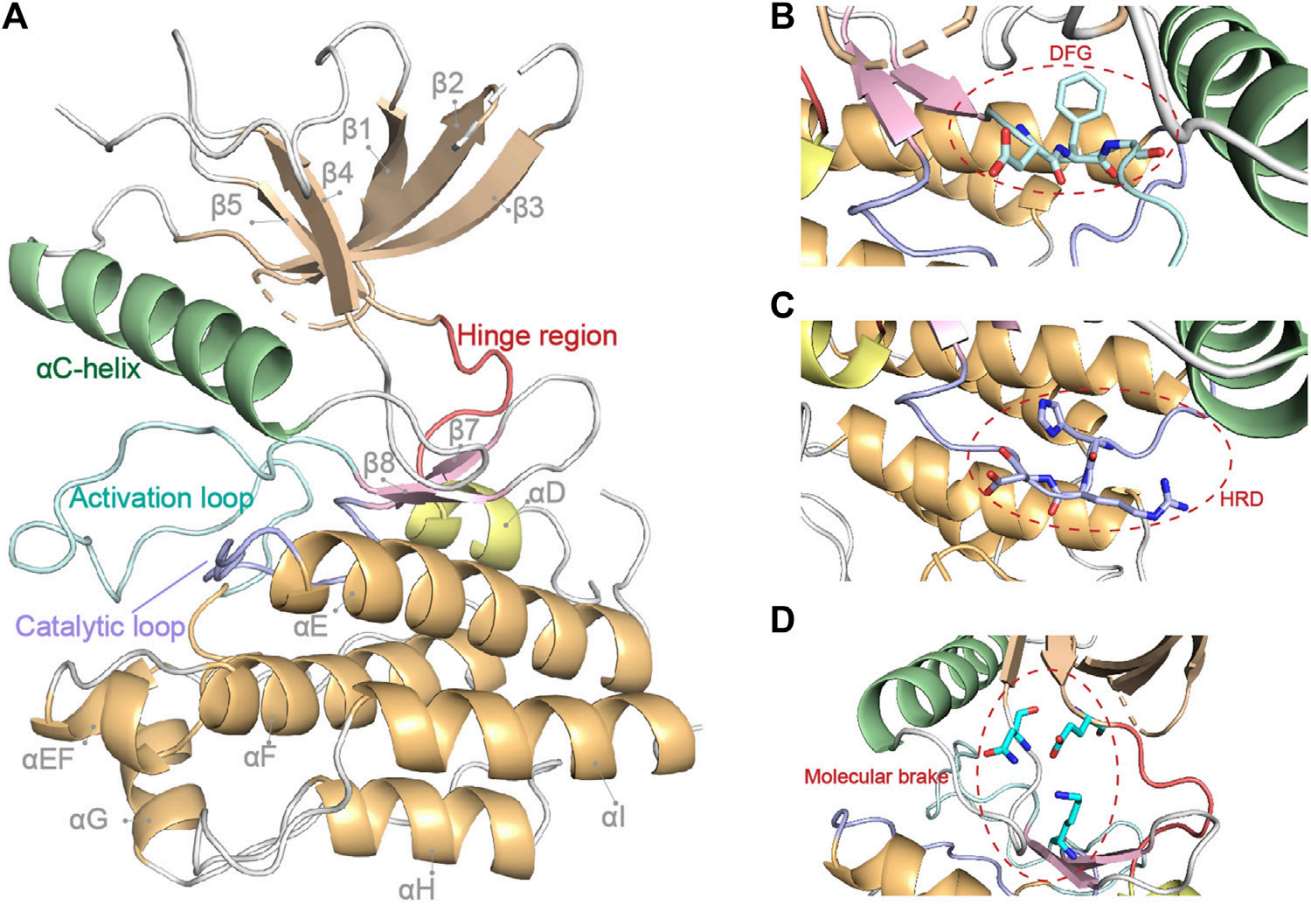
FGFR1 kinase domain structure (PDB: 4UWY). Basic secondary structures and critical regions described in this review are highlighted in **(A)**. The critical DFG, HRD motif and molecular brake are highlighted by close-up in **(B–D)**, respectively.

The intracellular tyrosine kinase domain of FGFR1-4 (∼300 amino acids) is the most extensively investigated part, which possesses a classical kinase architecture (Figure 2) (Itoh et al., 2004; Mohammadi, Schlessinger, et al., 1996; Sanchez-Heras et al., 2006). The small N-terminal lobe (N-lobe, ∼100 amino acids) is composed of a five-stranded anti-parallel β-sheet (*β*1-*β*5) and an *α*C-helix that resides between *β*3 and *β*4 in sequence and flanks the *β*-sheet spatially. A highly-flexible glycine-rich loop between *β*1 and *β*2 termed P-loop is able to swing downward in the presence of ATP to create the nucleotide binding site (Guimarães et al., 2011). In contrast, seven *α*-helices (*α*D, *α*E, *α*EF, *α*F, *α*G, *α*H and *α*I) gather to form the main part of the larger C-terminal lobe (C-lobe, ∼200 amino acids).

The N-lobe and C-lobe are connected by a hinge region containing conserved residues which could offer critical contacts with the adenine moiety of an ATP molecule. In addition, a triad of residues around the hinge region (e.g., N549, E565, and K641 in FGFR2) acts as the “molecular brake” of FGFRs to regulate their autoinhibition (Chen et al., 2007). The C-lobe also contains two long loops and some short strands at the interface of the two lobes, all of which contribute to the exquisite machinery for the activation and functioning of the kinase.

In between *β*8 and *α*EF, 20–30 amino acids round up to form the activation loop (A-loop), which encompasses tyrosine phosphorylation sites (Webster et al., 1996). At the beginning of the A-loop, an Asp-Phe-Gly triad constitutes the highly conserved DFG-motif, which is indicative of the active/inactive states of kinase. Located between *α*E and *β*7 is another important loop named catalytic loop, which contains the His-Arg-Asp (HRD) motif. The Asp of HRD-motif interacts with the hydroxyl group of the substrate tyrosine and therefore contributes to the phosphorylation (Vijayan et al., 2015).

### 3.2 FGF/FGFR Signaling

#### 3.2.1 Activation of FGFRs

Once FGFs bind to the extracellular domains of FGFRs, the dimerization of transmembrane and intracellular domains takes place along with a series of conformational changes that lead to trans-phosphorylation of dimerized kinase domains for activation.

The activation of kinase domain is a fined-tuned process (Furdui et al., 2006). Several critical tyrosine residues including Y463, Y583, Y585, Y653, Y654, Y730, and Y766 are autophosphorylated by precisely ordered reactions while ATP binds to the highly conserved pocket located in the hinge region during the activation of FGFR1 (Mohammadi, Dikic, et al., 1996). The autophosphorylations of Y653 and Y654 in the A-loop, which appear to induce the binding of substrate but not ATP, have increased by 50–100 fold and 500–1,000 fold in the rate of substrate phosphorylation, respectively. This suggested that these autophosphorylations have an indispensable role in kinase activation. The function of other tyrosine autophosphorylation sites contributes to the activation of FGFRs and downstream signal transduction through diverse biochemical reactions yet to be discovered.

In addition to the phosphorylation of critical tyrosine residues, the DFG motif of kinase domain toggles between two different conformations in line with the state of FGFR (active or inactive). When the motif adopts a DFG-in conformation, its Asp coordinates with phosphate groups of ATP and/or magnesium ion and causes the A-loop to display an open conformation, rendering the kinase an active state. Conversely, a DFG-out conformation, where the Asp and Phe point away from and toward the ATP binding pocket, respectively, is an indicator of inactive state of kinase. Noticeably, the flipped conformation of the DFG motif results in the formation of a large adjacent hydrophobic pocket (Hu et al., 2015; Vijayan et al., 2015). A valine in the ATP binding pocket (V561/564/555/550 in FGFR1/2/3/4), which is highly conserved in a variety of kinases and known as a “gatekeeper” residue, is the switch of the large hydrophobic pocket. The gatekeeper mutations give rise to many drug resistances due to hampered drug binding (Azam et al., 2008; Roskoski, 2010; Vijayan et al., 2015).

#### 3.2.2 FGFR Signaling Pathways

**FIGURE 3:**
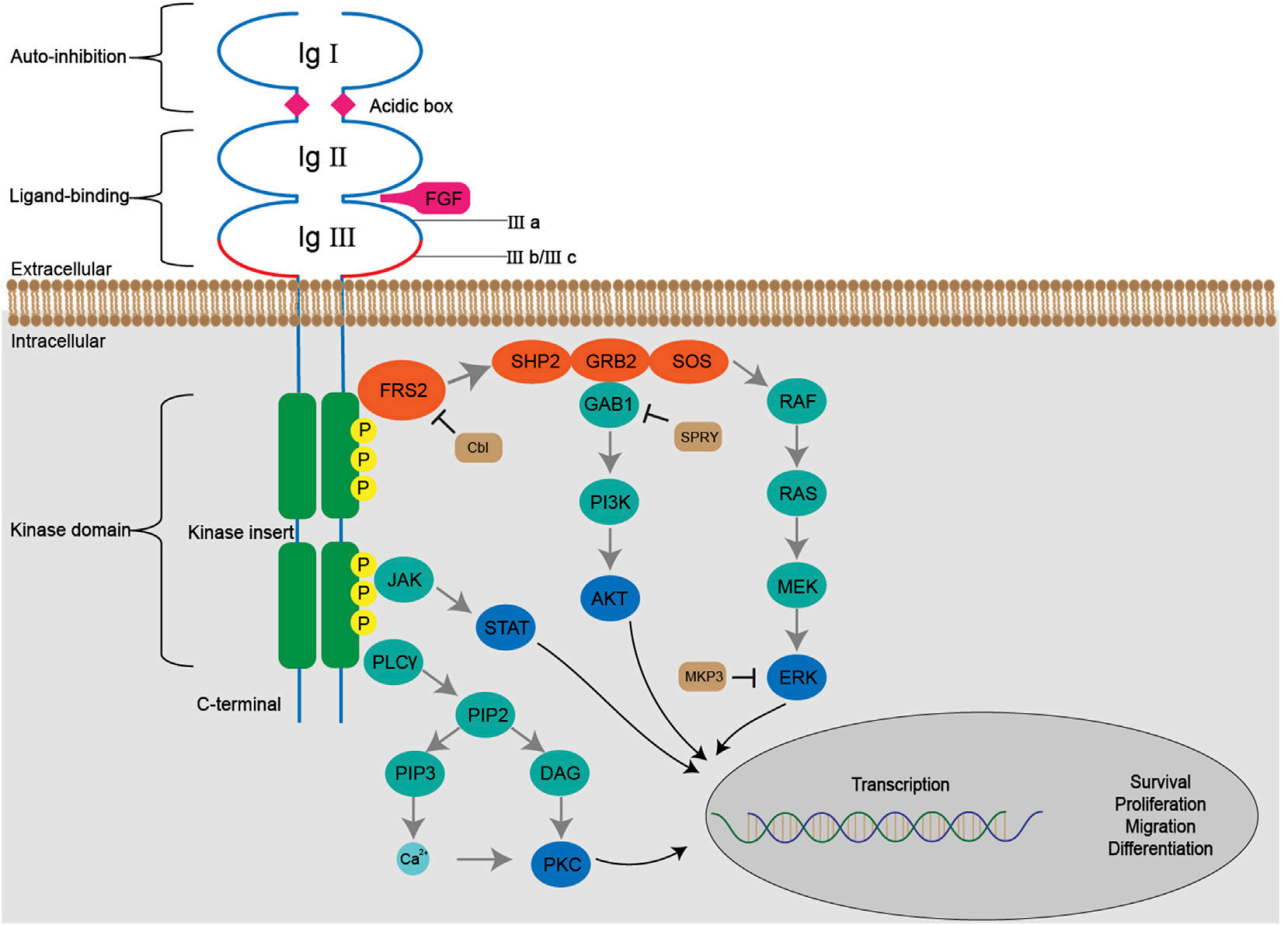
The FGF/FGFR signaling pathways. The binding of FGFs with FGFRs initiates a series of conformational changes, which consequently result in phosphorylation of tyrosine residues in the kinase domain. The phosphorylated tyrosine triggers cascaded docking and phosphorylation of downstream molecules including SHP2, GRB2, GAB1 and SOS, forms a multi-complex, and subsequently activates RAS-MAPK-ERK and PI3K-AKT pathways. Activated FGFRs are also involved in JAK-STAT and PLCγ-PKC pathways. The Cbl, SPRY, MKP3 negatively regulate FGF/FGFR signaling by ubiquitination, docking prevention and dephosphorylation, respectively.

The autophosphorylated kinase domain can recruit and phosphorylate multiple downstream effector molecules to initiate several signaling pathways (Figure 3).

The Fibroblast Growth Factor Receptor Substrate 2 (FRS2), a major FGFR substrate, binds to the juxtamembrane region of FGFR *via* its phospho-tyrosine binding domain (PTB) in a constitutive manner, regardless of the activation and phosphorylation state of the kinase domain. Following the activation of FGFR, multiple tyrosine residues of FRS2 are subject to phosphorylation and serve as docking sites for subsequent molecules.

The RAS-MAPK-ERK signaling pathway is activated by a serial docking of FRS2 with multiple proteins, including SH2-containing protein tyrosine phosphatase (SHP2), growth factor receptor-bound protein 2 (GRB2), Son of Sevenless (SOS) and noted RAS. Both SHP2 and GRB2 contain a Src homology domain (SH2 domain), which can recognize and bind the phosphorylated tyrosine residues of FRS2 and GRB2. Therefore, the GRB2-SOS complex is recruited to FRS2 directly or through the formation of the SHP2-GRB2-SOS complex (Hadari et al., 1998; Ong et al., 2000). The complex, in turn, initiates a phosphorylation cascade in the RAS-MAPK-ERK signaling pathway. Upon activation, ERK1/2 is translocated from cytoplasm into nucleus and regulates the activity of diverse transcription factors to influence cell proliferation, differentiation and signal transduction, which makes it the most persuasive signaling molecules in this pathway for the evaluation of FGFR inhibitors (Guo et al., 2020).

The docking protein GRB2 associated binding protein 1 (GAB1) is recruited to the complex *via* binding to the SH3 domain of GRB2, which enables tyrosine phosphorylation on itself. Similarly, the phosphorylated tyrosine residues of GRB2 are captured by the phosphoinositide 3 kinase (PI3K) containing a SH2 domain, thus initiating the activation of PI3K-AKT signaling pathway. The downstream effector molecules of AKT vary, including the well-known mTOR, which is closely related to cell metabolism, transcription and so forth (Quan et al., 2020).

Besides FRS2, the phospholipase Cγ (PLCγ) binds to a phosphorylated tyrosine in the C-terminal of phosphorylated kinase domain, and hydrolyzes phosphatidylinositol 4,5-bisphosphate (PIP2) into two secondary messengers, inositol triphosphate (IP3) and diacyl glycerol (DAG). The binding between IP3 and its receptor on the endoplasmic reticulum leads to the release of Ca^2+^ from intracellular stores and thus increases Ca^2+^ concentration (Mikoshiba, 2007). When coordinated with Ca^2+^, DAG activates PKC signaling pathway, which causes crosstalk with RAS-MAPK pathway due to the competition between GRB2 and PLCγ to bind with FGFR (Fearon et al., 2014).

In addition, FGFR can activate the signal transducer and activator of transcription (STAT) proteins to partially mediate cell transformation (Hart et al., 2000).

The negative regulation of FGF/FGFR signaling includes dephosphorylation, ubiquitination and obstruction in a serial of docking. In response to FGF stimulation, an ubiquitin ligase called Casitas B-lineage lymphoma (Cbl) is recruited to the FRS2 (-SHP2)-GRB2-SOS complex and induces ubiquitination and subsequent degradation of FGFR and FRS2α (Wong et al., 2002). In addition, the binding of Sprouty to GRB2 can block the interaction between GRB2 and FRS2 or SHP2 so as to exert an inhibitory effect on downstream RAS-MAPK signaling (Hanafusa et al., 2002). The dual-specificity MAPK phosphatases 3 (MKP3) also inhibits RAS-MAPK signaling by dephosphorylating activated MAPK (Farooq et al., 2004).

The activations of these FGFR-dependent or related signaling pathways converge into the regulation of diverse cellular events and physical functions.

### 3.3 FGF/FGFR Signaling in Cancer

**FIGURE 4:**
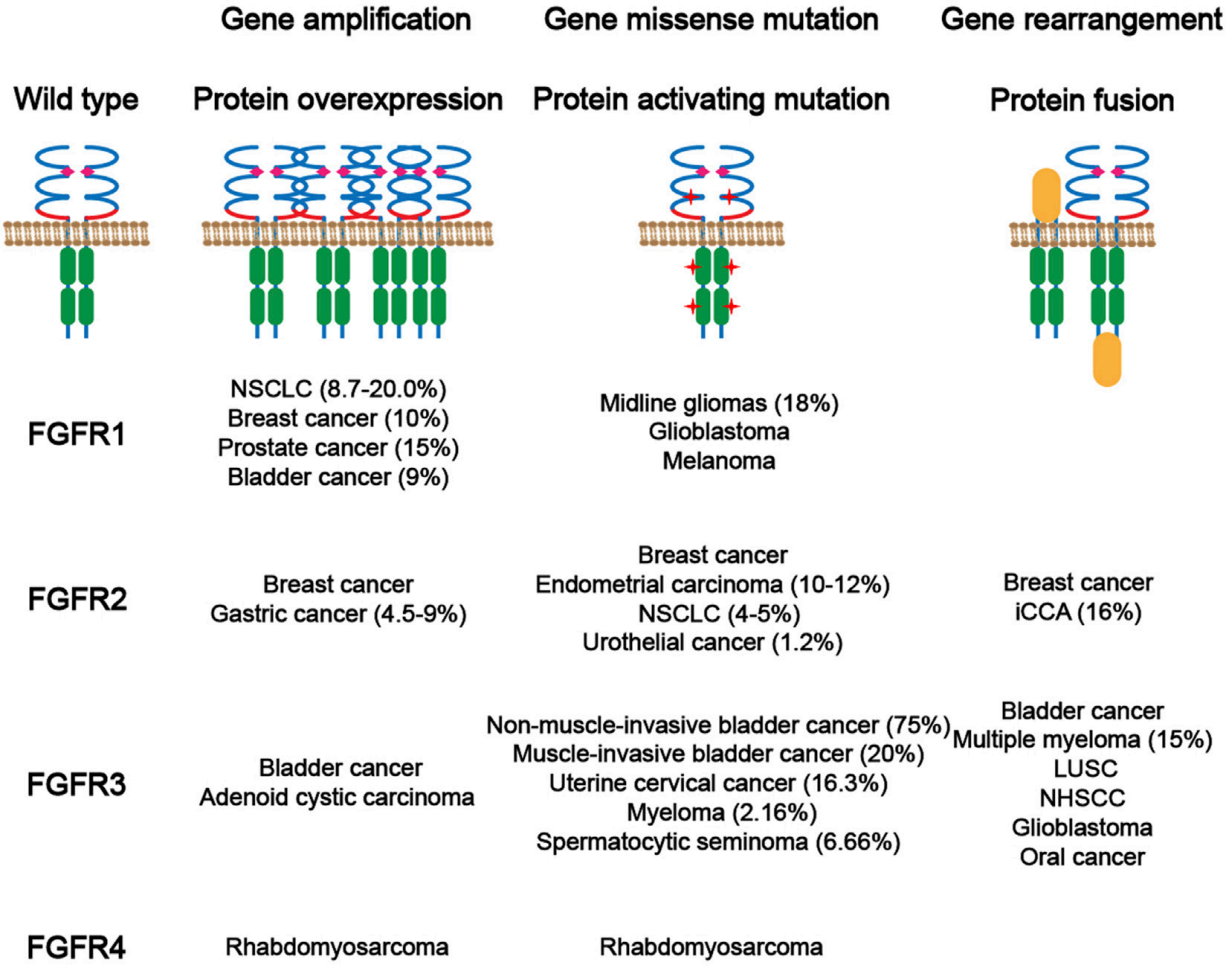
Main abnormalities in FGFRs and their frequency in related cancers.

FGFR genetic alterations have been involved in the development and progression of a variety of diseases, particularly cancers (Figure 4). The majority of FGFR aberrations are gene amplifications (66%), followed by gene mutations (26%) and, less frequently, rearrangements (8%), according to a recent sequencing study involving 4,853 patients with various types of cancers (Helsten et al., 2016). *FGFR* amplification leads to enhanced level of FGF binding. Generally, extracellular mutations increase binding affinity and disturb specificity between FGFs and FGFRs (Ibrahimi et al., 2001; Ibrahimi et al., 2004), or increase receptor dimerization by forming unexpected disulfide bridge (Plotnikov et al., 2000); while kinase domain mutations directly induce a higher level of intracellular phosphorylation. Despite the low incidence, chromosome rearrangements usually cause ligand-independent dimerization. However, most FGFR aberrations are oncogenic drivers, whereas prognostic indicators or “passenger co-aberrations” in different cancers remain ambiguous.

#### 3.3.1 FGFR1

As the most commonly altered FGFR subtype, FGFR1 aberrations account for 49% of all cases with FGFR aberrations according to the sequencing analysis (Helsten et al., 2016). The most frequent type of FGFR1 aberrations is gene amplification, which is reported in 8.7–20.0% of non-small cell lung carcinoma (NSCLC) cases (Miao et al., 2016; Miao et al., 2020) and involved in several acquired resistances against NSCLC therapeutics (Gammelgaard et al., 2019; Zhang et al., 2019). *FGFR1* amplification is common in breast cancer (10%), predominantly in estrogen receptor-positive breast cancer, and harmful to the survival of patients (Gelsi-Boyer et al., 2005; Wang et al., 2017). *FGFR1* amplification is also seen in prostate cancer (15%) (Edwards et al., 2003), bladder cancer (9%) (Ross et al., 2014), and other cancers (ovarian cancer, colorectal carcinoma, and squamous non-lung tumors). *FGFR* amplification (mainly in *FGFR1* and *2*) causes overexpression of proteins and increases the FGFR-dependency of cancer cells. Therefore, it is regarded as a biomarker for the efficacy of some FGFR inhibitors (Weiss et al., 2010). *FGFR1* mutation has been detected in several tpyes of cancers, including midline gliomas (18%), glioblastoma and melanoma, whereas *FGFR1* fusion is rare.

#### 3.3.2 FGFR2

Amplification (predominantly observed in triple-negative breast cancer, 4%) and mutation (e.g., K660N) of FGFR2 occur frequently in breast cancer. Besides, *FGFR2* also amplifies in gastric cancer (4.5–9%) and is associated with its venous and lymphatic invasion (Jung et al., 2012; Cancer Genome Atlas Research Network, 2014). Apart from breast cancer, 10–12% of endometrial carcinoma and 4–5% of NSCLCs bear *FGFR2* mutations (Mohammadi et al., 1998; Dutt et al., 2008; Kandoth et al., 2013). *FGFR2* mutants are infrequently reported in urothelial cancers (1.2%). Several *FGFR2* fusions have been reported including *FGFR2*-*AFF3*, *FGFR2*-*CASP7* and *FGFR2*-*CCDC6* (Turner et al., 2010; Reintjes et al., 2013; Wu et al., 2013). In addition, *FGFR2* fusions occur in cholangiocarcinoma, lung squamous cell carcinoma (LSCC), thyroid cancer, prostate cancer, according to a study of FGFR targetable gene fusions (Wu et al., 2013). Notably, a *FGFR2*-*PPHLN1* fusion generated by the chromosomal translocation t (10; 12) (q26; q12) is identified to possess oncogenic and transforming activity in 16% of intrahepatic cholangiocarcinoma (iCCA).

#### 3.3.3 FGFR3

FGFR3 aberrations are predominantly implicated in bladder cancer (Baldia et al., 2016; Helsten et al., 2016). The incidence of *FGFR3* mutations in non-muscle-invasive bladder cancer is as high as 75%, as determined by the presence of mutations in the p53 suppressor gene (Zhang et al., 2015), whereas it is relatively low (20%) in muscle-invasive bladder cancer (Couffignal et al., 2015; Solomon et al., 2016; Siracusano et al., 2020). Suppression of FGFR3 activation is sufficient to reduce the survival and proliferation of carcinoma cells harboring *FGFR3* mutations (Markham, 2019; Montazeri et al., 2020). Furthermore, *FGFR3* mutations are found in uterine cervical cancer (16.3%) (Yoshimoto et al., 2020), including invasive cervical cancer (5%) (Rosty et al., 2005), myeloma (2.16%) (Walker et al., 2015), and spermatocytic seminoma (6.66%) (Goriely et al., 2009). *FGFR3* amplification is not frequent in cancers, but is sporadically reported in bladder cancer and adenoid cystic carcinomas (Vékony et al., 2007). Translocations at the t (4; 14), in which FGFR3 is significantly mutated, occurs in multiple myeloma (15%) frequently (Walker et al., 2018). Fusions of *BAIAP2L1* or *TACC3* to 5’ terminal of *FGFR3* can also cause aberrant activation of FGFR3 by inducing oligomerization of fusion proteins even in the absence of FGFs. These fusions are reported in a variety of cancers including bladder cancer, LSCC, NLSCC, glioblastoma and oral cancer.

#### 3.3.4 FGFR4

Amplification or mutation of *FGFR4* is rarely perceived as an oncogene except in rhabdomyosarcoma (7.5%). It is confirmed that kinase inhibitor treatment increased cell apoptosis in *FGFR4*-mutant rhabdomyosarcoma (RMS) cell lines, which is consistent with increased SubG1 fraction and high level of activated caspase-3, suggesting the strong dependency of RMS on FGFR4 (Taylor et al., 2009).

## 4 Small-Molecule FGFR Inhibitors

To fight against FGFR-driven abnormalities in various cancers, continuous efforts are devoted to various types of therapeutics, including monoclonal antibodies interacting with extracellular domain of FGFR, ligand traps restricting FGF, and small-molecule inhibitors targeting the kinase domain. During the past decade, we have witnessed multiple preclinical and clinical breakthroughs of FGFR inhibitors. To help developing novel therapeutics, we reviewed the current status of discovery of small-molecule FGFR inhibitors as well as other small molecule-based modalities from the standing point of medicinal chemists.

Although the development of tyrosine kinase inhibitors started in the 20th century (Porta et al., 2017), targeting FGFR was validated as a therapeutic strategy for cancer treatment only recently, when FDA approved the use of erdafitinib (JNJ-42756493) in 2019 (Markham, 2019), pemigatinib (INCB054828) in 2020 (Qu et al., 2022) and infigratinib (BGJ-398) in 2021 (Yu et al., 2021) for the treatment of FGFR-altered cancers. In addition, a larger number of inhibitors are in clinical trial or preclinical investigation, such as LY2874455, ARQ-087, AZD4547, FGF401, BLU9931, and H3B6527s (Supplementary Table S1). The following part will elaborate the discovery of small-molecule FGFR inhibitors in structure-based fashion.

### 4.1 The First-Generation: Non-Selective FGFR Kinase Inhibitors

The FGFR kinase domain share high homology with other receptor tyrosine kinases. The first-generation FGFR inhibitors are non-selective tyrosine kinase inhibitors (TKIs) that compete with ATP for ATP-binding site. As a result, these inhibitors inhibit not only FGFR but also a variety of other tyrosine kinases, such as vascular endothelial growth factor receptor (VEGFR), platelet-derived growth factor receptor (PDGFR), fms-like tyrosine kinase 3 (FLT-3), c-Kit and BCR-ABL (Huang et al., 2020).

**FIGURE 5:**
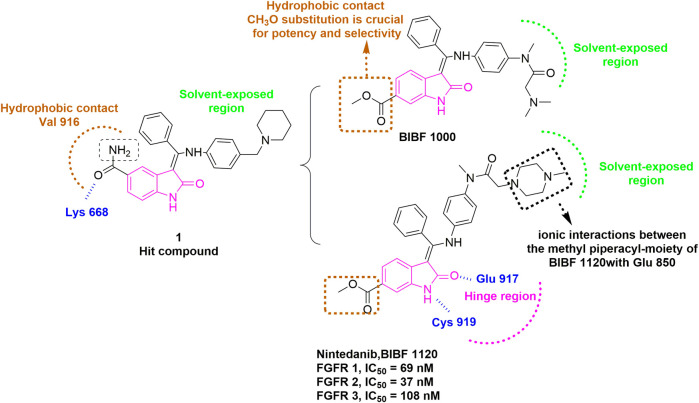
Discovery of BIBF1120. H-bonds are outlined as blue hashed lines. The methyl-piperazinyl moiety is involved in the ionic interaction with the side chains of Glu850 (Black). Hydrophobic interaction is outlined by hashed brown rectangle. Solvent-exposed region is highlighted using green rectangle. Hinge region is indicated by pink arc.

Many approved TKIs show mild to strong activity for FGFR, and some of them are being (or have been) assessed in clinical trials for diseases where FGFR alterations are implicated, including nintedanib, dovitinib, ponatinib, lucitanib, derazantinib, anlotinib, and so on. Nintedanib (BIBF1120), first discovered in 2009 by Roth et al. (2009), is an inhibitor targeting VEGFR, FGFR and PDGFR (Capdevila et al., 2014). Nintedanib was approved for the treatment of idiopathic pulmonary fibrosis and interstitial lung diseases (ILD) by FDA in 2014 and 2020, respectively. It is currently under active clinical trials, including the treatment of *FGFR3* mutated urothelial carcinoma (Phase 2, NCT02278978), and the treatment of SARS-CoV-2 induced pulmonary fibrosis (Phase 3, NCT04541680). This inhibitor resulted from the optimization of a hit compound **1** bearing a 5-substituted indolinone core that was initially identified as a VEGFR-2 inhibitor (Figure 5). The computational modeling of hit compound **1** with VEGFR-2 suggested that the carbonyl oxygen of the amide group can form a hydrogen bond with Lys868. The hydrophobic region flanked by Val916 indicated that replacing the amide moiety with a more lipophilic substituent (e.g., methoxycarbonyl) could improve potency and maintain selectivity. Meanwhile, the basic side chain pointing toward the solvent was further modified with additional polar fragments, resulting in two compounds BIBF1000 and BIBF1120. The latter compound exhibited a favorable IC_50_ values for VEGFR, FGFR, and PDGFR within nanomolar range and showed selectivity over other homologous kinases. The indolinone scaffold formed two hydrogen bonds with Cys919 and Glu917 in the hinge region. The methyl piperazinyl group directed into the solvent region, and its 4-nitrogen atom formed a bidentate ionic interaction with the carboxylate oxygens of Glu850 based on a published X-ray crystal structure in complex with VEGFR-2 (PDB: 3C7Q) (Hilberg et al., 2008).

Dovitinib (TKI258) inhibits VEGFR-2, FGFR-1, and PDGFR with IC_50_ values below 0.1 μM, and several clinical trials for advanced solid tumors have been conducted (Angevin et al., 2013; Taeger et al., 2011). Dovitinib contains a benzimidazole core, which makes critical contacts with the hinge region and also binds to FGFR1 and FGFR4 in a DFG-in mode as usually observed for this type of inhibitors (Bunney et al., 2015; Lesca et al., 2014). Ponatinib, which targets PDGFR, VEGFR and FGFR, was initially approved for the treatment of refractory chronic myeloid leukemia (CML) or Philadelphia chromosome-positive acute lymphoblastic leukemia (Ph + ALL) in 2012 (Pao et al., 2004; Cortes et al., 2018), then it entered two clinical trials in 2014 for the treatment of malignant neoplasm with *FGFR* fusions or activating mutations (NCT02265341, NCT02272998). Structural study revealed that ponatinib bound to either FGFR1 or FGFR4 in a unique DFG-out mode, which is distinct from most of reported FGFR inhibitors (Lesca et al., 2014). Liu et al. (2017) conducted extensive SAR study of ponatinib and obtained optimized analogs with improved activity and selectivity. Anlotinib, a quinoline-based inhibitor of VEGFR, FGFR, PDGFR and c-kit (Shen et al., 2018), is being investigated for treatment of advanced solid tumors with FGFR alterations (NCT03929965). Lucitanib (E3810) is also a TKI that targets VEGFR1/2/3, FGFR1/2 and PDGFR (Babina et al., 2017), and a phase 2 trial (NCT02053636) for testing Lucitanib in patients with *FGFR1*-amplified or non-amplified ER + metastatic breast cancer was completed. Derazantinib (ARQ087) inhibits multiple kinases including RET, DDR2, PDGFR, VEGFR, KIT and FGFR, and its phase 1/2 study in FGFR-altered patients was recently completed as well. Representative kinase small-molecule inhibitors in this category are shown in Supplementary Figure S1 *.*

Although moderate suppression on tumors harboring FGFR aberrations was observed, these non-selective inhibitors still brought some issues of therapeutic regimen. The human kinome comprises ∼535 protein kinases (Zhong et al., 2021). A wide range of off-target effects attributed to their poor selectivity leads to blockage of multiple signaling pathways and causes a multiplicity of related side effects such as diarrhea, vomiting and nausea (Konecny et al., 2015). Albeit these factors have restricted the broad application of multi-target TKIs, they are widely recognized as a decent treatment for tumors in absence or unawareness of the “oncogenic driver,” and have provided the impetus to the development of on target FGFR inhibitors.

### 4.2 The Second-Generation: Selective FGFR Kinase Inhibitors

Thanks to the rapidly evolving high throughput screening methods and structure-based strategies, a number of second-generation FGFR inhibitors have been discovered with higher potency, selectivity, safety as well as novel modality. Three inhibitors in this category, namely erdafitinib, pemigatinib, and infigratinib, have been approved by FDA, and a lot more compounds are being evaluated in preclinical and clinical investigations. These second-generation inhibitors were tentatively divided into several subclasses on the basis of different modes of action. Chemical structures of these reported selective FGFR small molecule inhibitors are shown in Supplementary Figure S2 *.*

#### 4.2.1 Non-Covalent Pan-FGFR Inhibitors

The three approved FGFR inhibitors and quite a few candidates are all non-covalent inhibitors with pan-FGFR inhibitory activity, although some of them showed reduced, yet still considerable, potency for FGFR4 because of its relatively notable difference from FGFR1-3.

**FIGURE 6:**
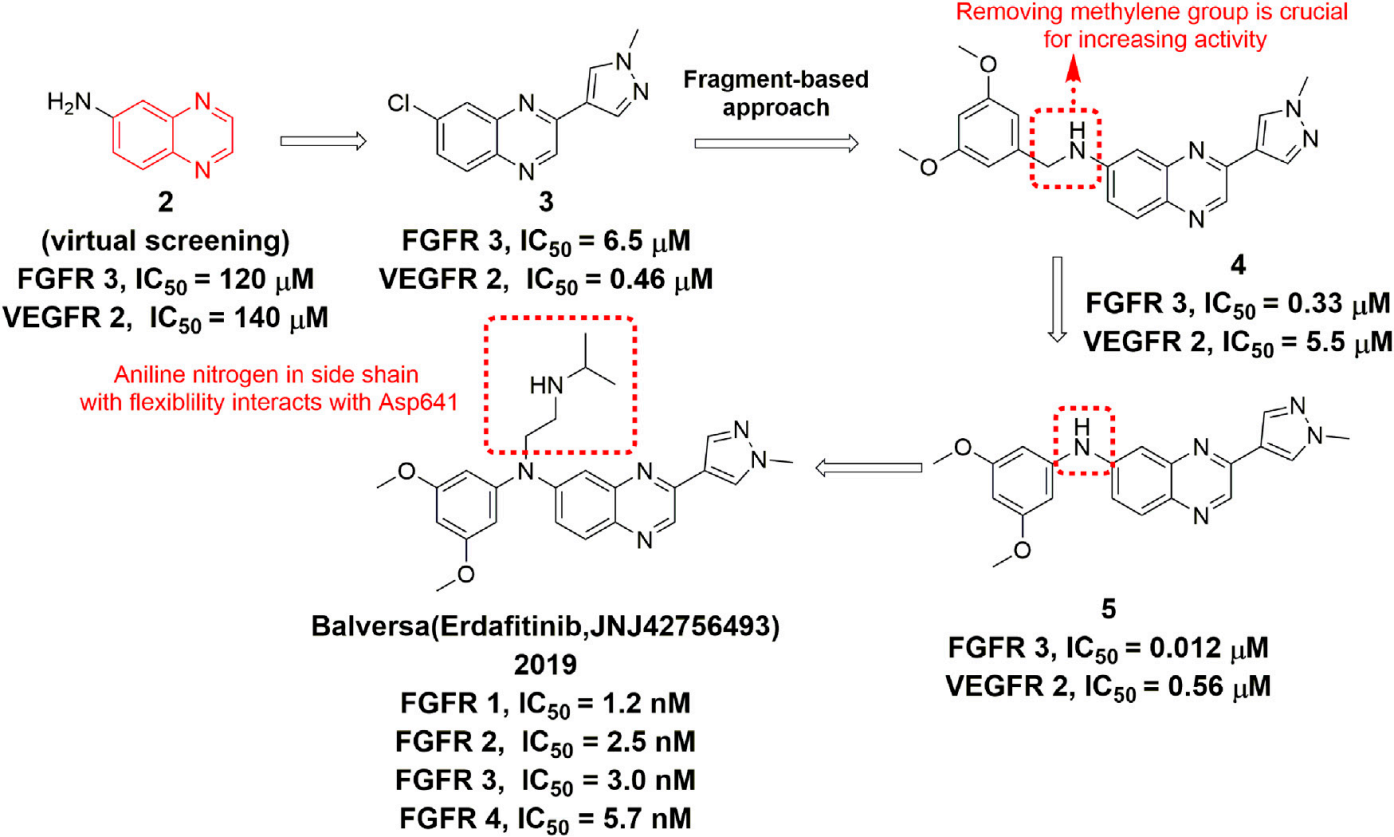
Discovery of erdafitinib.

Erdafitinib (JNJ-42756493) is the first approved FGFR inhibitor for treatment of adult patients with locally advanced or metastatic urothelial carcinoma. It is an orally active and selective pan-FGFR inhibitor (Perera et al., 2017) that inhibits the kinase activity of FGFR1-4 with similar potency (Markham, 2019). Erdafitinib features quinoxaline element, which was first identified through virtual screening based on the crystal structure with FGFR1. The compound **2** was next generated through fragment growing approach. Removal of the methylene group in compound **2** produced compound **3**, which has shown much improvement in activity due to better shape complementarity with the hydrophobic pocket. An additional substitution on the secondary nitrogen occupied the ribose-binding region, leading to the discovery of erdafitinib with increased affinity, better physicochemical and pharmacokinetic properties (Figure 6 **)** (Murray et al., 2019).

**FIGURE 7:**
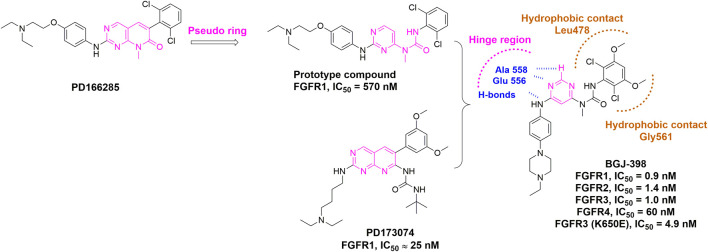
Discovery of infigratinib (BGJ-398) and summary of interactions of infigratinib and FGFR3 ATP binding site. H-bonds with hinge region are indicated by blue hashed lines. Hydrophobic interactions are outlined by hashed brown rectangle (PDB: 3TT0).

In order to replace the pyrido [2,3-*d*]pyrimidin-7-one core, which was a common feature in a well-established class of protein kinase inhibitors (e.g., PD166285), Guagnano et al. (2011) developed a pseudo six-membered ring structure stabilized by an intramolecular hydrogen bond (e.g., prototype compound) (Figure 7) (Furet et al., 2008). Using the same strategy, infigratinib was eventually discovered through a lead optimization of a known FGFR inhibitor PD173074*.* Structural studies confirmed that hydrogen bonds with hinge region were retained and both chlorines and methoxy groups would form favorable hydrophobic contacts with the deep pocket inside ATP binding site. It was also observed that the phenyl ring at the entrance of the pocket was hydrophobically sandwiched between Leu478 and Gly561. These hydrophobic effects contributed to the selectivity of infigratinib for FGFR, especially FGFR1-3, over other tyrosine kinases. Infigratinib was approved by FDA for the treatment of cholangiocarcinoma patients with FGFR2 fusion in 2021 (Botrus et al., 2021; Javle et al., 2021).

**FIGURE 8:**
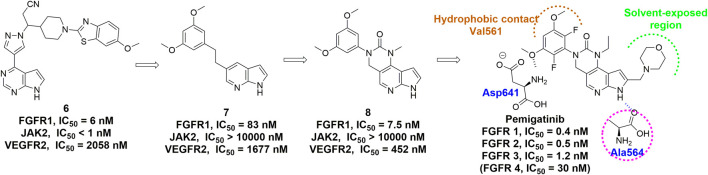
Discovery of pemigatinib and summary of interactions of pemigatinib and FGFR1 ATP binding site. H-bonds are indicated by blue hashed lines. Hydrophobic interactions are outlined by hashed brown arc. Solvent-exposed region is highlighted using green arc.

Pemigatinib is another FGFR inhibitor featuring a tricyclic urea scaffold for the treatment of adults with previously treated, unresectable locally advanced or metastatic cholangiocarcinoma with a FGFR2 fusion or other rearrangement. Like infigratinib, pemigatinib contains a 3,5-dimethoxyphenyl for the high affinity and selectivity by filling the hydrophobic pocket, and two fluorine atoms, which led to further improvements in potency (Figure 8) (Wu et al., 2021). Many rounds of optimization also demonstrated that the ethyl group on the N-1 position of the cyclic urea and the pyrrole ring were of great importance to the potency and PK profile.

**FIGURE 9:**
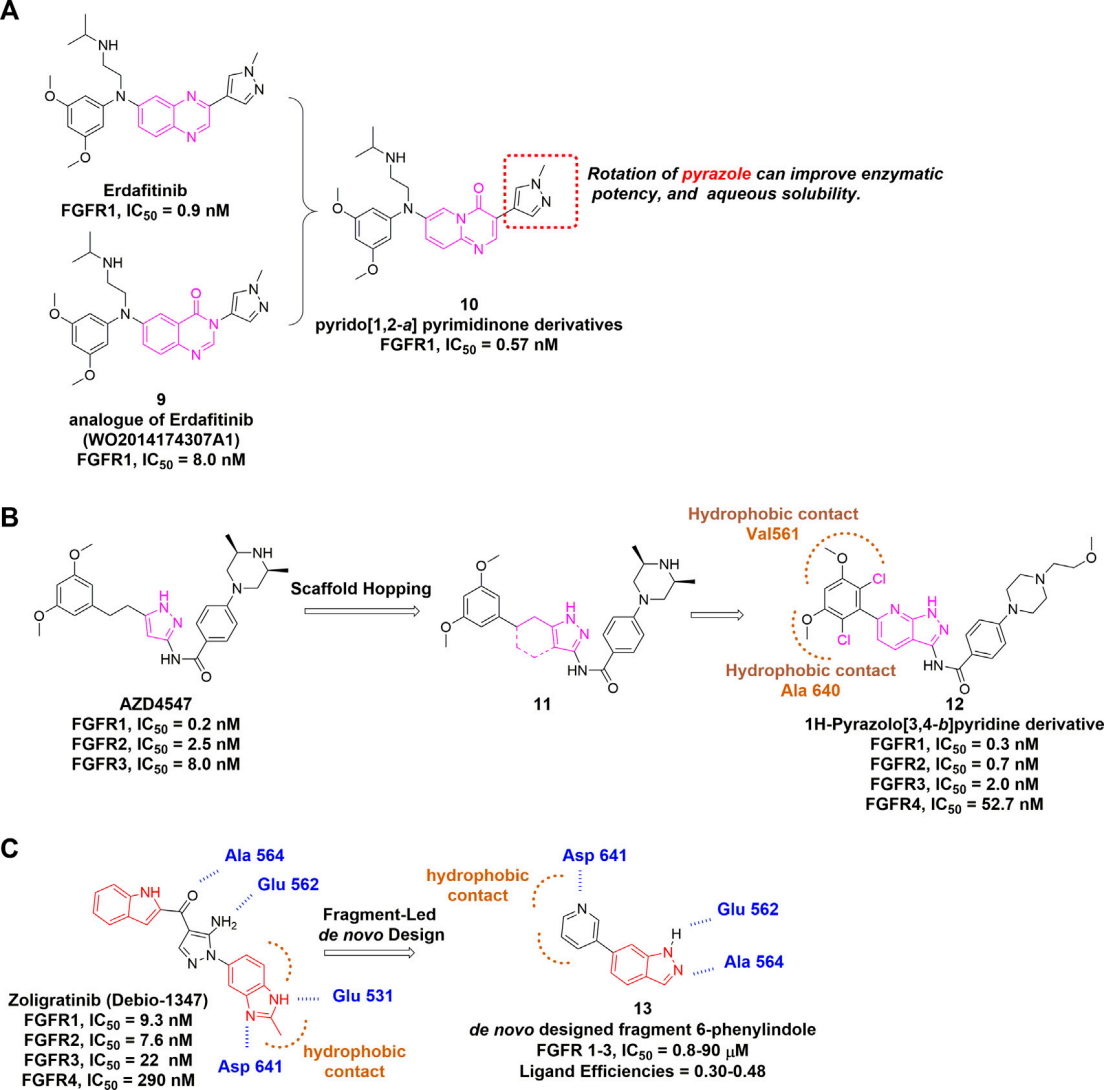
**(A)** Discovery of pyrido [1,2-*a*] pyrimidinone derivatives as non-covalent pan-FGFR inhibitor. **(B)** Discovery of 1H-Pyrazolo [3,4-*b*]pyridine derivatives from AZD4547 and summary of its interactions with FGFR1 ATP binding site. Hydrophobic interactions are outlined by hashed brown arc (PDB: 4V05). **(C)** Summary of CH5183284 and designed fragment.

Starting from erdafitinib and its quinazolinone analogue, a series of pyrido [1,2-*a*] pyrimidinone derivatives were designed as novel selective FGFR inhibitors through scaffold hopping (Figure 9A) (Ran et al., 2021). Molecular docking suggested an overall similar binding mode with erdafitinib, while the rotatable pyrazole ring could lead to increased potency. The rotation also disrupted the planarity, which might enhance the aqueous solubility owing to reduced crystal-stacking.

AZD4547 is another pan-FGFR inhibitor bearing the pyrazole scaffold and just completed phase 2 clinical trial (NCT04439240) in multiple cancers with FGFR alterations. Based on the structure of AZD4547, Zhao *et al.* developed 1H-Pyrazolo [3,4-*b*]pyridine derivatives using scaffold hopping strategy, and incorporated two chlorines at the dimethoxyphenyl ring (Figure 9B) (Zhao et al., 2016). Notably, AZD4547 maintains efficacy to FGFR1 harboring gatekeeper mutation V561M as the flexible linker between dimethoxyphenyl and pyrazole allows conformational adaption within the hydrophobic region (Sohl et al., 2015). However, it remains unknown whether 1H-Pyrazolo [3,4-*b*]pyridine derivative (**a**) has compromised its activity for mutated FGFRs.

CH5183284 (Debio1374) is a potent pan-inhibitor of FGFR1-3 with IC_50_ values of 9.3, 7.6 and 22 nM, respectively. It was discovered through a conventional high throughput screening and its interactions with the hinge region, hydrogen bonding pattern and an additional π-π interaction were also identified (Ebiike et al., 2016). CH5183284 is under clinical investigation for the treatment of cancer patients with FGFR genetic alterations (Nakanishi et al., 2014). Turner et al. (2017) described the application of SPROUT, a *de novo* computation program, to develop the active indazole-based pharmacophore for the inhibition of FGFR kinases **(** Figure 9C **)**. Beginning with the co-crystal structure of CH5183284-FGFR1, they modified the indole moiety and obtained a small library of 23 indazole derivatives. Subsequent biological evaluation indicated that these indazole-containing fragments inhibited FGFR1-3 with IC_50_ values of 0.8–90 µM, suggesting that structure-based drug discovery (SBDD) is becoming an essential tool for identifying potent and selective FGFR inhibitors.

There are numerous other non-covalent pan-FGFR inhibitors in clinical trial or development. For example, ASP5878 inhibited cell proliferation of urothelial cancer cell lines harboring FGFR3 point mutation or fusion and has completed phase 1 clinical trial in 2017 (NCT02038673) (Kikuchi et al., 2017). LY2874455 is a phase 1 orally available inhibitor with IC_50_ values of 2.8, 2.6, 6.4, and 6.0 nM against FGFR1-4, respectively (Michael et al., 2017). Rogaratinib (BAY 1163877) is another potent and selective FGFR1-4 inhibitor (Collin et al., 2018). Rogaratinib alone or in combination with other agents have been in a few clinical trials. 3D185, a highly selective FGFR1-3 inhibitor, was approved for investigational new drug by the NMPA in 2018 and followed by a phase 1 study in patients with the advanced solid tumors (NCT04221204). ICP-192 is a pan-inhibitor against FGFR1-4 and entered the phase 1/2 clinical trial for the treatment of advanced solid tumors, urothelial carcinoma, and cholangiocarcinoma (NCT04565275). E7090 has favorable pharmacokinetic profiles and sub-nanomolar inhibitory activity against FGFR1-3 with IC_50_ values of 0.71, 0.50, and 1.2 nM, respectively (Watanabe Miyano et al., 2016). The phase 2 study of E7090 in participants with unresectable advanced or metastatic cholangiocarcinoma with *FGFR2* fusion is recruiting (NCT04238715).

#### 4.2.2 Covalent Pan-FGFR Inhibitors

Covalent inhibition is a re-emerging strategy especially in the development of kinase inhibitors, which can make a big difference in binding affinity and selectivity. A covalent inhibitor typically consists of a drug-like scaffold offering noncovalent interactions and an appropriate electrophilic warhead to react with nucleophilic residues of target proteins. Cysteine represents the most targeted amino acid in kinases, due to its non-catalytic roles, low abundance, high reactivity and chemical plasticity of the anionic thiolate (Abdeldayem et al., 2020; Galbiati et al., 2020). For FGFRs, the conserved cysteine in the P-loop (C488 in FGFR1, C491 in FGFR2, C482 in FGFR3 and C477 in FGFR4) and the unique C552 in FGFR4 in the hinge region are sites for covalent attachment (Dai et al., 2019). This section focuses on recent publications with regard to the discovery of covalent pan-FGFR covalent inhibitors.

Zhou et al. (2010) discovered FIIN-1 as the first irreversible inhibitor of FGFR1−4 in 2010. The acrylamide of FIIN-1 formed covalent bond with a conserved cysteine (Cys488 of FGFR1) located at the rim of the P-loop. Replacing the acrylamide with a propylamide led to the failure of covalent bond formation. In addition, its selectivity over some other kinases (e.g., c-Src, TNK1, and YES) bearing the P-loop cysteine at the same position as FGFRs was also confirmed. Tan et al. (2014) developed FIIN-2 and FIIN-3 as irreversible inhibitors with potent *in vitro* inhibitory activity against FGFR1 and FGFR2 gatekeeper mutants, which conferred resistance to first-generation FGFR inhibitors. The acrylamide moiety in both molecules was installed on the 4-poistion of their phenyl rings in contrast to 3-acrylamide as found in FIIN-1, which still maintained the bond formation with P-loop cysteine while changed the selectivity over other kinases (PDB 4QQC, 4R5S, 4R6V).

**FIGURE 10:**
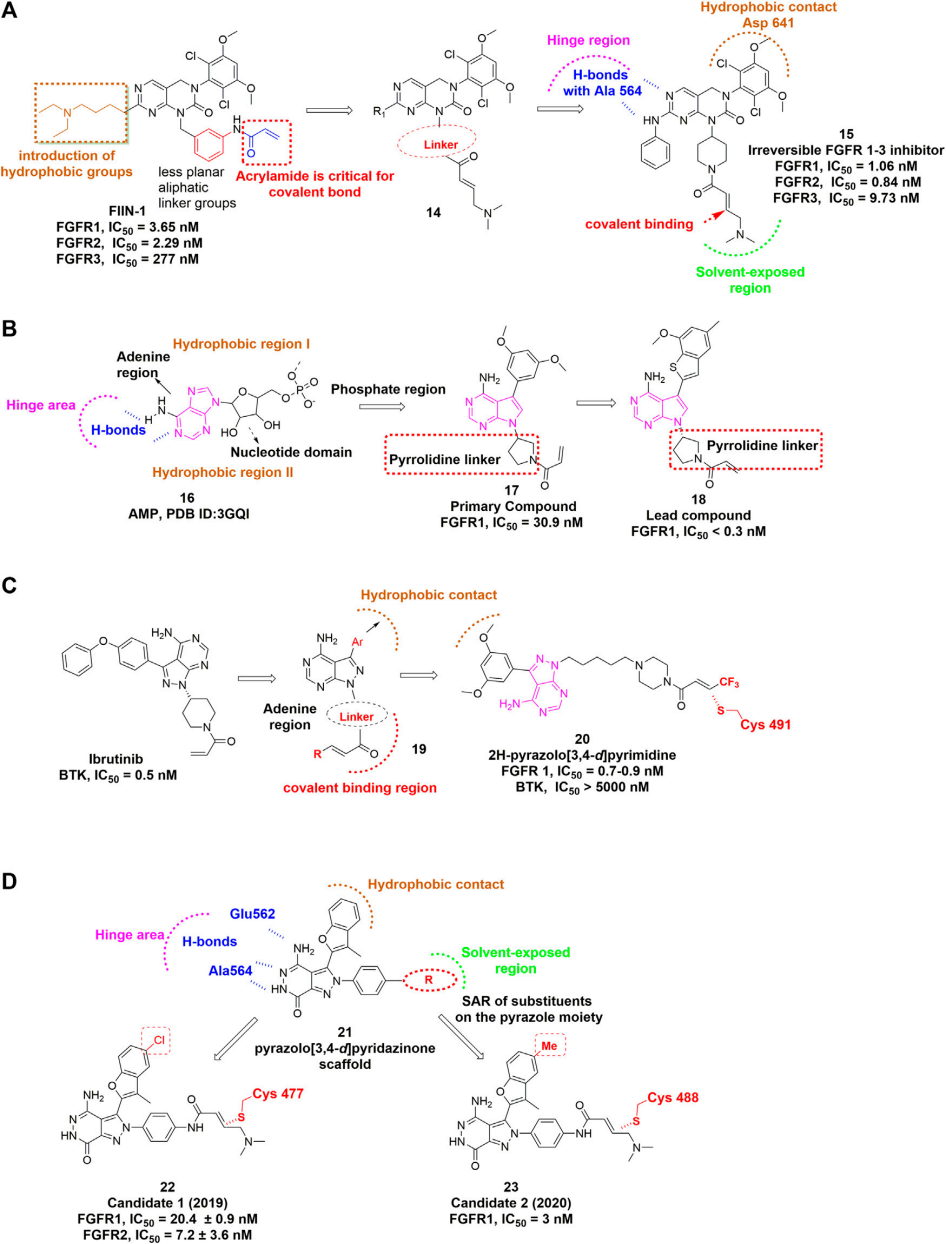
**(A)** Structure optimization of FIIN-1. H-bonds are indicated by blue hashed lines. Hydrophobic interactions are outlined by hashed brown arc (PDB: 5B7V). The covalent bond-forming Michael acceptor carbon of the acrylamide is indicated by red arrow. **(B)** Discovery of irreversible inhibitors bearing novel pyrrolopyrimidine scaffold. **(C)** Discovery of 2H-pyrazolo [3,4-*d*]pyrimidine derivatives. Hydrophobic interaction region is outlined by hashed brown arc. Covalent binding region is highlighted by dashed red arc. **(D)** Covalent FGFR inhibitors bearing pyrazolo [3,4-*d*]pyridazinone.

Brameld et al. (2017) developed another irreversible inhibitor, PRN-1371, which shared a common core with FIIN-1. PRN-1371 was proven to be highly selective for FGFR1-4 over other kinases, including KDR, FLT-4, etc., and showed high FGFR1 occupancy and ideal PK profile. Ding *et al.* focused on the modification of the acrylamide-containing side chain of FIIN-1 and obtained the promising lead compound showing inhibitory effect in FGFR-amplified cancer cell lines **(** Figure 10A) (Li et al., 2017). The crystal structure of FGFR1 C488A in complex with lead compound revealed that the acrylamide side chain was located in the solvent accessible space and easily performed nucleophilic attack by the target cysteine.

Goyal et al. (2019) demonstrated that Futibatinib (TAS-120), a highly selective and irreversible pan-FGFR inhibitor, exhibited *in vitro* potency against wild-type FGFR1-4, as well as some FGFR2 kinase domain mutants. Hiroshi Sootome et al. (2020) reported the preclinical profile of futibatinib and suggested that it is an orally available, potent pan-FGFR inhibitor. Futibatinib is the most advanced candidate in the category of covalent pan-FGFR inhibitors because it is in phase 3 clinical trial to evaluate the treatment of metastatic and recurrent unresectable cholangiocarcinoma harboring FGFR2 gene rearrangements.

Wang et al. (2018) designed and discovered a set of irreversible inhibitors bearing novel pyrrolopyrimidine scaffold. By analyzing the ATP binding pocket, they introduced a substituted phenyl moiety at the C-3 position of pyrrolopyrimidine to interact with hydrophobic region I **(** Figure 10B **)**. Then the electrophilic warhead attached to N-1 *via* a pyrrolidine linker led to the identification of lead compound that showed excellent potencies against FGFR1−4 and acceptable selectivity over VEGFR2.

Wei et al. (2021) recently reported a class of 2H-pyrazolo [3,4-*d*]pyrimidine derivatives as a potent irreversible pan-FGFR inhibitor (Figure 10C). The lead compound was derived from BTK inhibitor ibrutinib and also shares a similar core with the above-mentioned lead compound. Interestingly, an electron-withdrawing terminal-CF_3_ substituted acrylamide group provided the most potent inhibition against FGFRs.

There are several other covalent pan-FGFR inhibitors in development. For example, Yamani et al. (2021) discovered a pyrazole-benzimidazole CPL304110 as a pan-FGFR inhibitor for the treatment of bladder, gastric and squamous cell lung cancer, which also showed favorable pharmacokinetic properties after oral administration. Dai et al. (2020) reported that DW14383 simultaneously inhibited tumor proliferation and angiogenesis *via* inhibition of FGFR1–4 with similar potency. In addition, they claimed that its pan-tumor spectrum potential might overcome compensatory activation among FGFR1–4. Wang et al. (2019) developed a series of compounds featuring pyrazolo [3,4-*d*] pyridazinone as covalent FGFR inhibitors. Their structural optimization resulted in more analogues that could remarkably inhibit proliferation of various FGFR-dysregulated cancer cells and display potent antitumor efficacy in xenograft model as well (Figure 10D) (Xie et al., 2020).

#### 4.2.3 FGFR4-Specific Covalent Inhibitors

**FIGURE 11:**
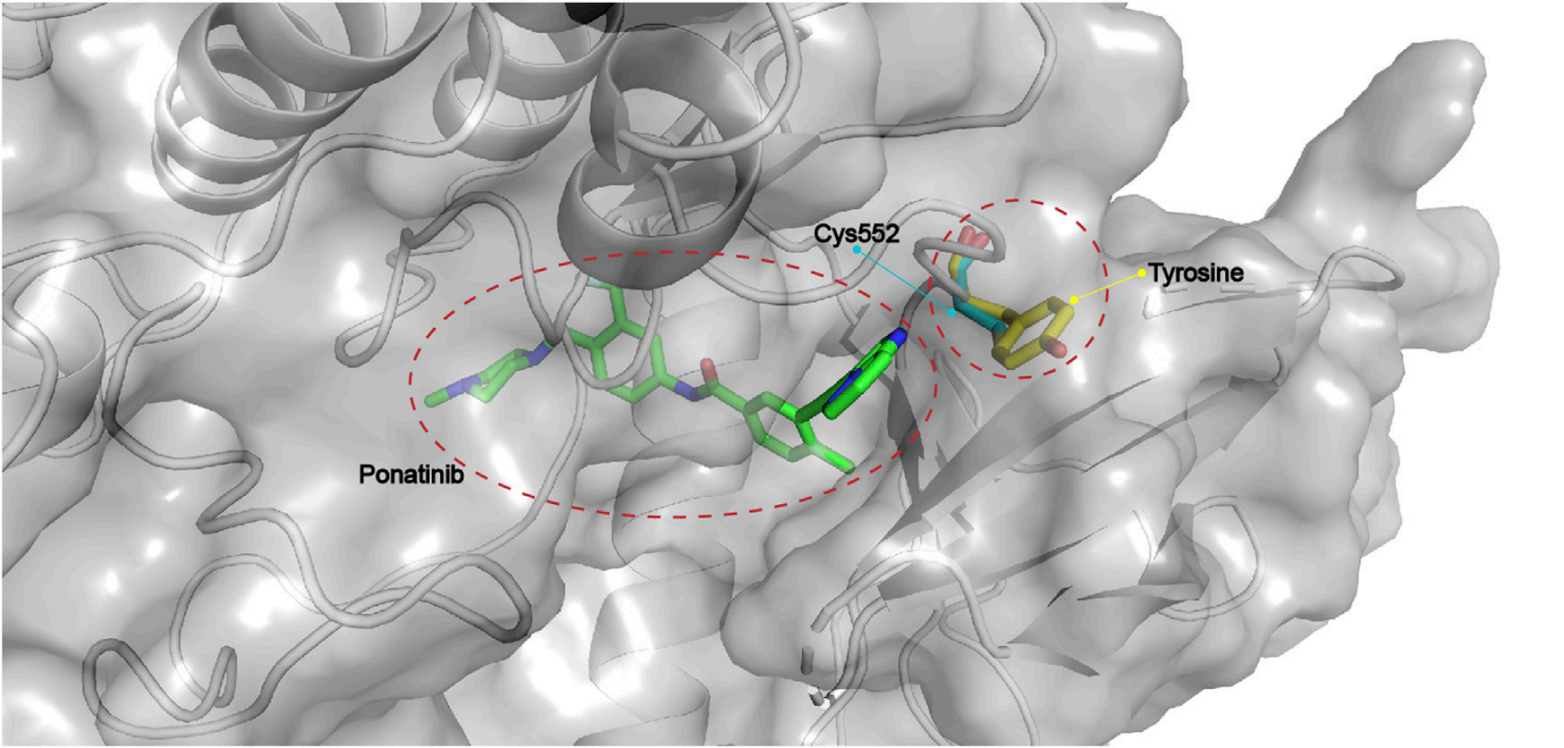
Critical Cys552 in FGFR4 and replacement by tyrosine in FGFR1-3 (PDB: 4QRC, the crystal structure of FGFR4 in complex with Ponatinib).

The kinase domains of FGFR1–3 share high structural similarity, whereas FGFR4 is relatively distinct from FGFR1-3 (Babina et al., 2017), which is consistent with the fact that many foregoing pan-FGFR inhibitors show strong inhibition of FGFR1-3 but reduced potency for FGFR4. Detailed comparison of the active sites of FGFR1-3 and FGFR4 revealed a key difference in the hinge region: Tyr563 in FGFR1-3 versus the Cys552 in FGFR4 **(** Figure 11 **)** (Tucker et al., 2014). This unique Cys552 provides great opportunity for the design of highly selective, covalent inhibitors of FGFR4. Although there is no approved FGFR4-specific drug, the past years have witnessed growing numbers of promising compounds as discussed below.

**FIGURE 12:**
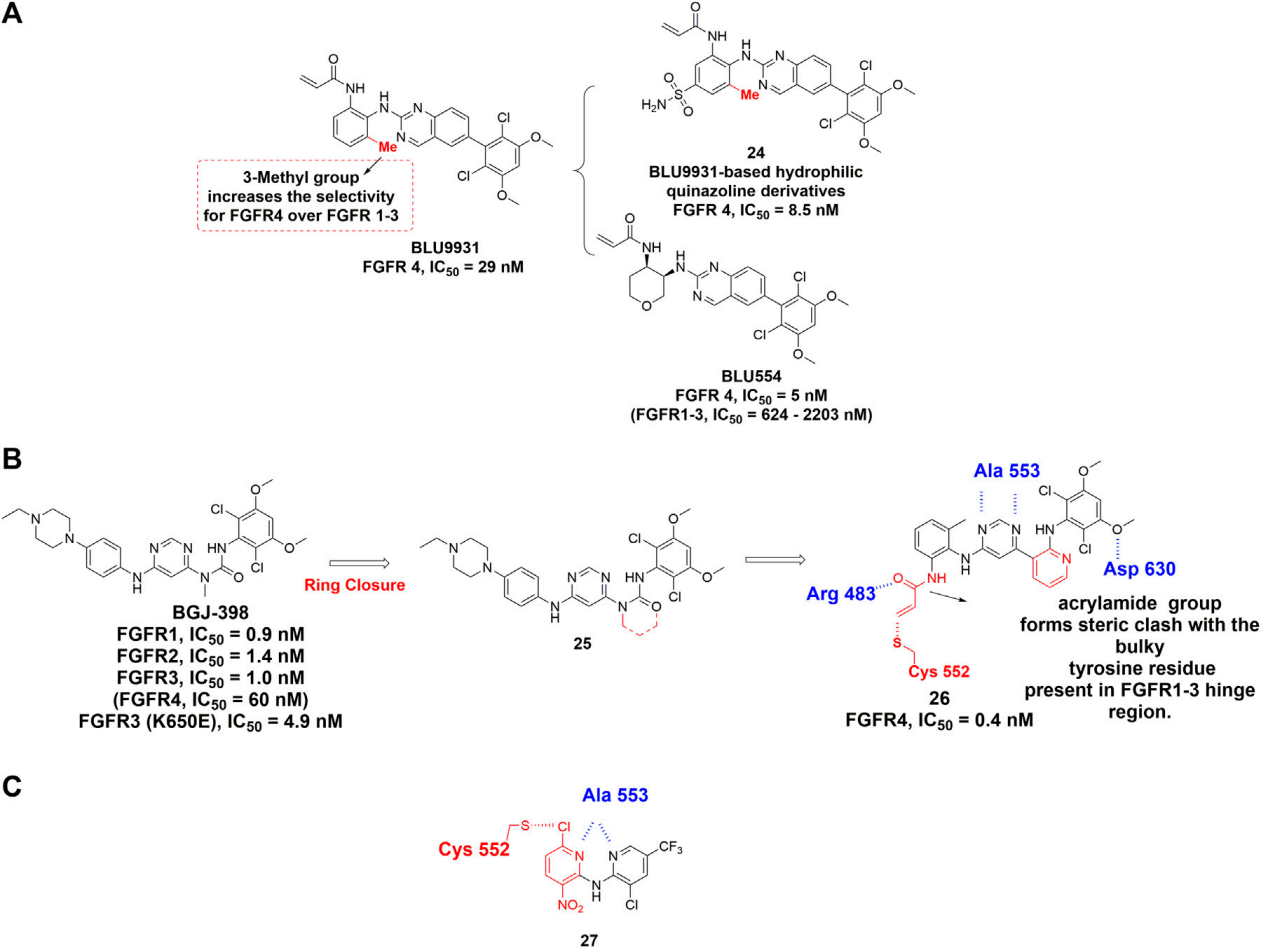
**(A)** Structures of FGFR4 covalent inhibitors BLU9931 and its analogs. **(B)** Discovery of a covalent inhibitor of FGFR4 from BGJ-398 and summary of its interactions with FGFR 4 kinase domain. H-bonds are outlined by hashed blue line. Covalent binding contact is highlighted using red (PDB: 6JPE). **(C)** Discovery of dipyridylamine. The novel warhead is highlighted by red color and H-bonds are outlined by hashed blue line.

Hagel et al. (2015) discovered BLU9931 as the first selective FGFR4 inhibitor for the treatment of hepatocellular carcinomas (HCC) with aberrant FGFR4 signaling. The dimethoxyphenyl group of BLU9931 occupied the hydrophobic pocket located near the gatekeeper valine of all FGFRs. In addition, an acrylamide at the ortho-position of the aniline can form the covalent bond with Cys552 in the hinge region of FGFR4. To achieve a better selectivity, the rotation of the phenyl ring was also taken into consideration because it could cause steric clash with the corresponding tyrosine in FGFR1-3 hinge region. Moreover, the addition of 3-methyl group on the aniline ring rendered BLU9931 with high selectivity for FGFR4 over FGFR1-3 (Figure 12A). Unfortunately, BLU9931 failed to enter clinical stage, presumably due to its rapid metabolism in liver microsomes. BLU554 (fisogatinib), an orally available analog of BLU9931, is now in phase 1 clinical trial to treat patients with HCC (NCT02508467) and in phase 1b/2 clinical trial in combination with CS1001.

H3B-6527 (Joshi et al., 2017) is another highly selective covalent FGFR4 inhibitor and is currently undergoing phase 1 study for the treatment of advanced HCC, liver neoplasms, hepatic carcinoma and so on. Co-crystal structure of FGFR4 in complex with H3B-6527 illustrated the covalent bond formation between Cys552 and the acrylamide at the ortho-position of the N-aryl substituent.

Fairhurst et al. (2020) reported the discovery of FGF401 (roblitinib) as a potent, selective FGFR4 inhibitor. Through high throughput screening, 2-formylquinoline amide (2-FQA) derivatives were identified as the starting hits. After optimization of the 2-FQA and the substituent groups on the hinge-binding pyridyl ring, roblitinib was eventually obtained with outstanding selectivity for FGFR4. Being assessed in phase 2 clinical trial, roblitinib is the most advanced covalent FGFR4-specific inhibitor. This inhibitor features a covalent yet rapidly reversible mode of action that may reduce off-target related toxicity. Therefore, roblitinib is regarded as a promising next-generation drug to offer a new approach to target FGFR covalently.

Starting from BGJ-398 (infigratinib), Miranda et al. developed a novel covalent inhibitor of FGFR4 for the treatment of HCC (Rezende Miranda et al., 2020). According to the crystal structure of FGFR1-BGJ-398, the urea group and aminopyridine group should exhibit similar geometrical and electronic properties **(** Figure 12B). An acrylamide group was also attached to the ortho position of a 2-methylaniline ring for targeting Cys552 of FGFR4. Interestingly, crystallographic study revealed that the introduction of the methyl group into aniline phenyl rings facilitates the covalent reaction from a conformational perspective (PDB: 6JPE). As expected, its exceptional selectivity among the FGFR family is due to the fact that the acrylamide group formed steric clash with the bulky tyrosine residue present in FGFR1-3 hinge region.

Fairhurst et al. (2017) reported the discovery of dipyridylamine through high throughput screening (HTS). Dipyridylamine contains a novel warhead 3-nitro-6-chloropyridyl, which was designed to achieve covalent binding with Cys552 of FGFR4 (Figure 12C **)**. The 6-chloro substituent in this warhead is positioned for attack by the Cys552 thiolmethyl group through a nucleophilic aromatic substitution (S_N_Ar). Dipyridylamine demonstrated high potency against FGFR4 with IC_50_ value of 53 nM while sparing the FGFR1-3 with IC_50_ values higher than 10 μM. Besides, each nitrogen atom in pyridyl ring formed a hydrogen bond with hinge residue Ala553 (Lu et al., 2019). Dipyridylamine features a relatively low molecular weight and novel mechanism of covalent binding that may serve as a promising lead compound for future discovery of FGFR4-specific covalent inhibitors.

Several candidates have entered clinical stages without full disclosure of chemical structures. For example, INCB-62079 entered phase 1 trial in 2017 but was terminated for business strategic consideration. ICP-105, a selective FGFR4 inhibitor, is now in phase 1 clinical trial for the treatment of solid tumor (NCT03642834). Other clinical trials involving inhibitors including ZSP-1241 and ABSK-011 are actively recruiting patients.

#### 4.2.4 Selective FGFR2 Inhibitors

**FIGURE 13:**
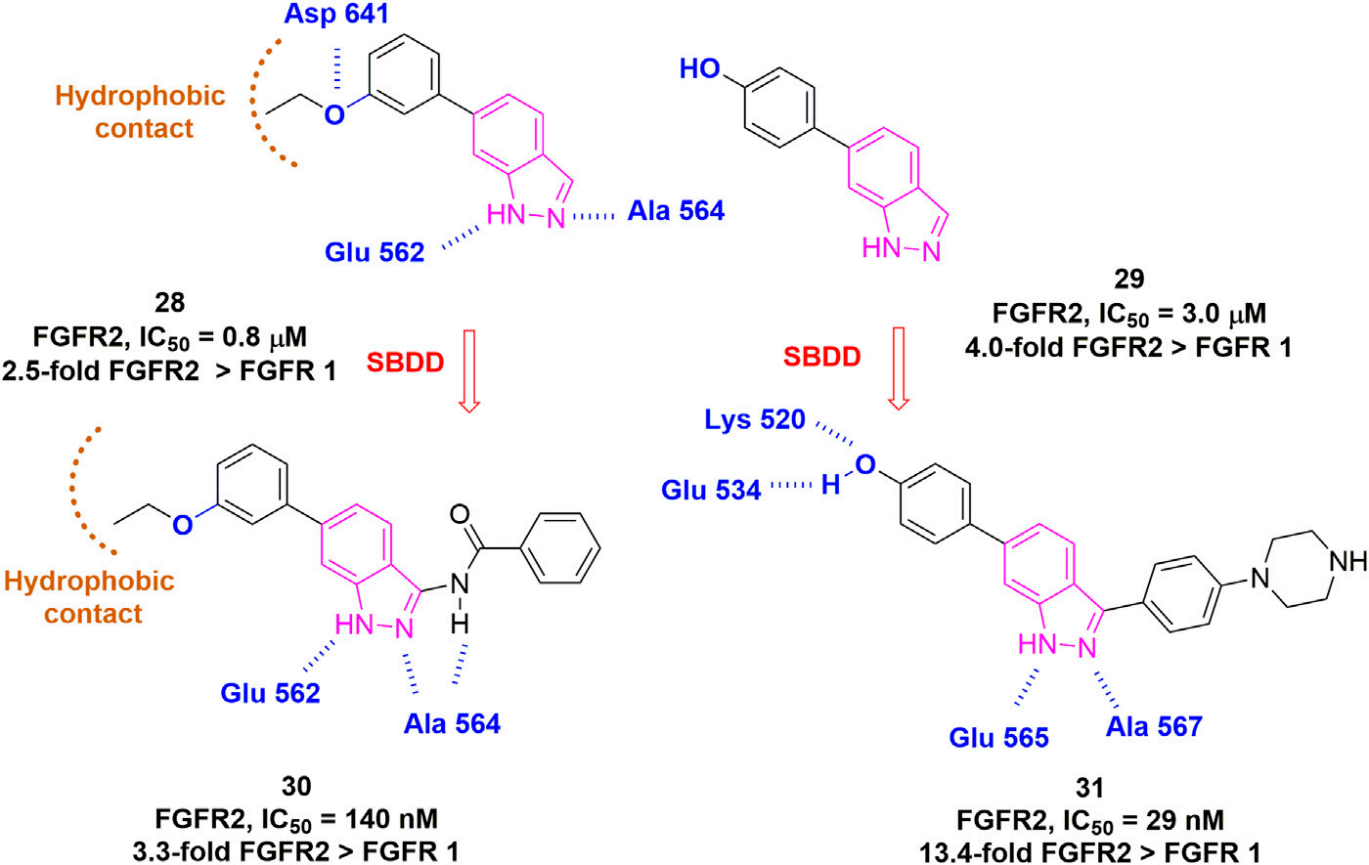
Structure-guided approach for the development of selective FGFR2 inhibitors. H-bonds are outlined as blue hashed lines. Hydrophobic region is indicated by brown arc.

Unlike FGFR4, few FGFR1-3 subtype-specific kinase inhibitors have been reported to date, mainly aiming at FGFR2. Casaletto et al. (2021) recently reported RLY-4008 as a highly selective inhibitor of FGFR2 WT/mutant, which exhibited >200-fold higher potency than FGFR1. Although the structure has not been disclosed, RLY-4008 showed no difference in binding modes with FGFR1 or FGFR2. Instead, a flexible loop in FGFR1/2 validated from MD simulation might be the cause of the selectivity. It is encouraging that a recent *de novo* design by Turner et al. (2021) has provided a paradigm for perhaps the next-generation member of FGFR-specific inhibitors. They started from a fragment with moderate potency and carried out iterative rounds of *de novo* design as well as a classical SAR study to generate compound **31**. Interestingly, compound **31** specifically inhibited FGFR2 with an IC_50_ of 29 nM, whereas 389 nM for FGFR1 and 758 nM for FGFR3, suggesting at least 10-fold selectivity for FGFR2 over FGFR1 (Figure 13).

### 4.3 Miscellaneous Types

#### 4.3.1 Extracellular Allosteric Inhibitors

An extracellular allosteric inhibitor of FGFR can bind to its extracellular domain and modulate the receptor conformation, thus blocking the signaling pathway. The extracellular domains of FGFR1-4 exhibit discernible structural differences compared to their kinase domains, therefore it is possible to achieve selective inhibition within FGFR members. Alofanib (RPT835) belongs to this type of inhibitors and has entered a phase 1 clinical trial in Russia. By specifically binding to IgIII of FGFR2, alofanib is able to inhibit the FGF2-induced phosphorylation of FRS2α with nanomolar activity in cancer cells expressing different FGFR2 isoforms (Tyulyandina et al., 2017). In addition, Tsimafeyeu et al. (2016) performed *in vivo* experiments to demonstrate that alofanib could ablate FGF-induced angiogenesis. Another example is SSR128129E, an orally-active, allosteric inhibitor of FGFR1 (Herbert et al., 2013). It interacts with extracellular part of FGFR without interfering with FGF binding or receptor dimerization. Critical conformational changes were observed in IgIII subdomain when treated with SSR128129E, resulting in defective internalization of FGFRs.

#### 4.3.2 Natural Products

Efforts have been made to identify natural products that act on FGFRs. These include a few phenolic compounds (resveratrol, caffeic acid phenethyl ester, kaempferol, etc.), stilbene glycosides, and sesterterpenes compounds (leucosesterterpenone, leucosterlactone, etc.), and a detailed review has been completed by Yin et al. (2019). Recently, Pagano et al. (2021) reported rosmarinic acid (RA), a natural phenolic compound, could induce FGF2/FGFR complex dissociation as verified by experimental mechanistic study. In addition, gambogenic acid and ferulic acid, originated from natural plants, exhibited inhibitory effect of FGFR autophosphorylation. Taken together, natural products stand for a prime source of FGFR inhibitors, while more studies are needed to improve their activities and elucidate the molecular mechanisms. Chemical structures of these represent natural products are shown in Supplementary Figure S3 *.*

## 5 Other Small Molecule-Based Therapeutic Modalities

### 5.1 Combination Therapy

Due to the extensive crosstalk between FGF/FGFR and other signaling pathways, the inhibition of FGF/FGFR signaling can be rescued by activation or upregulation of multiple signaling pathways. The most involved proteins are among the receptor tyrosine kinase family, such as c-Met, EGFR, ErbB2/3 or even among four members of FGFR. The compensatory activation of alternative receptors or/and signaling pathways occurs frequently while a receptor function is suppressed specifically, and consequently the resistance to FGFR inhibitor is developed. Therefore, a combination of FGFR inhibitor with other types of drugs is a promising avenue to improve clinical efficacy of available therapeutics and overcome drug resistance.

Fischer et al. (2008) reported that cotreatment with FGFR inhibitors (SU5404/PD166866) and EGFR-targeting drugs (erlotinib/lapatinib) improved *in vivo* antiproliferative effects, indicating its potential as combination therapy in NSCLC. In addition to EGFR, other RTKs were also involved in the combination therapy with FGFR. The combined treatment of RET inhibitor (ST1571) and FGFR inhibitor (PD173074) significantly suppressed tumor growth of medullary thyroid cancer, which is intractable by surgery and has no widely accepted treatment (Ezzat et al., 2005). The addition of VEGFR1 inhibitors solved the limited practical effects of FGFR inhibitors in FGFR1-amplified breast cancers through blocking the contribution of FGFR1 to VEGF secretion (Golfmann et al., 2018).

Besides RTKs, many kinases are attractive targets in the combination therapy with FGFR inhibitors. The phosphoinositide 3-kinase (PI3K) inhibitor is used to achieve superior antitumor effect in FGFR2 mutant endometrial cancer cell lines (Packer et al., 2017). PI3K also mediates resistance to FGFR inhibitors in urothelial cell carcinomas harboring alterations of FGFR3 gene, which both highlight the prospect of combination of their inhibitors (Wang et al., 2017). FGFR was also identified as a promoter to induce resistance to CDK4/6 inhibitors, which was diminished by complementary inhibition of FGFR in ER+/FGFR1-amplified breast cancers (Formisano et al., 2019). Similar antitumor effects were observed in synergism of mTOR and FGFR inhibitors, which resulted in significantly arrested cell cycle in G1 phase in AN3CA-derived endometrial tumor models (Gozgit et al., 2013). Krook et al. (2020) further implemented a combination therapy using an mTOR inhibitor (INK258) and demonstrated that this strategy may overcome the resistance to FGFR inhibitor like infigratinib. Moreover, through a kinome-wide CRISPR-based screening, Yang et al. identified PLK1 and FGFR as promising synthetic lethal targets for treating FGFR1-amplified lung cancer (Yang et al., 2021).

Overexpression of FGFs may also cause hyperactivated FGF/FGFR signaling, which is present in some tumors and can be co-targeted accordingly. The frequent presence of both BRAF mutations and FGF2 overexpression in melanomas, which lack a recognized systematic therapy so far, leads to the combination of FGFR inhibitor PD166866 and BRAF V600E inhibitor, consequently increased cell apoptosis and restricted tumor growth (Metzner et al., 2011). Wang et al. (2019) further revealed that upregulated secretion of FGF1 gave rise to resistance to the combined therapy of RAF inhibitor vemurafenib and MEK inhibitor cobimetinib in BRAF V600E-driven tumors, which was abrogated by addition of FGFR inhibitors to achieve a triple BRAF/MEK/FGFR inhibition.

Tremendous potential also lies in cooperation of FGFR inhibitors with immune checkpoint inhibitors (Qin et al., 2020). For instance, Palakurthi *et al.* demonstrated the combination of erdafitinib and PD-1 blockade RMP1-14 could achieve remarkable tumor regression and significantly improve survival in mice with a FGFR2-driven lung tumor harboring dual mutations on FGFR2 and P53 genes (Palakurthi et al., 2019).

Despite a number of successful attempts of combination therapy, the drug-drug interactions may cause unpredictable toxicity and should be assessed with meticulousness. For example, the combination of infigratinib with imatinib encountered higher toxicity and frequent adverse effects, including CPK elevation, lipase elevation, hyperphosphatemia, anemia, and peripheral edema (NCT02257541).

### 5.2 Dual/Multi-Target Inhibitors

Dual/multi-target inhibitors have several potential advantages over combination therapy, such as more predictable pharmacokinetics, better patient compliance, reduced administration dosage and toxicities (Duan et al., 2021). Previous advances in the field of TKIs validated a diversity of promising and well-tolerated targets, including EGFR, ALK, ROS1, HER2, NTRK, VEGFR, RET, MET, MEK, FGFR, PDGFR, PI3K and KIT, which have inspired the discovery and rational design of dual/multi-target inhibitors.

From the perspective of medicinal chemistry, most of the present dual/multi-target inhibitors can be assigned to the first- or second-generation FGFR TKIs. In some cases, their low selectivity over aforementioned targets has in turn created a synergistic inhibitory effect in diseases involving abnormal FGFR and the other target(s). MPT0L145, an alleged dual-target inhibitor of PIK3C3 and FGFR, not only increased autophagosome formation due to FGFR inhibition but also interfered with autophagic flux via PIK3C3 inhibition, It synergistically sensitized anticancer effects of targeted- or chemo-therapy in different cancer cell lines (Chen et al., 2018). Besides, FGFR/EGFR and FGFR/VEGFR dual inhibition strategies are also frequently reported. FGFR/EGFR dual inhibitors can be exemplified by FIIN3, while FGFR/VEGFR dual inhibitors include PD173074, AZD2171 (cediranib), BMS-540215 (brivanib), ODM-203, and so on (Chen et al., 2019).

**FIGURE 14:**
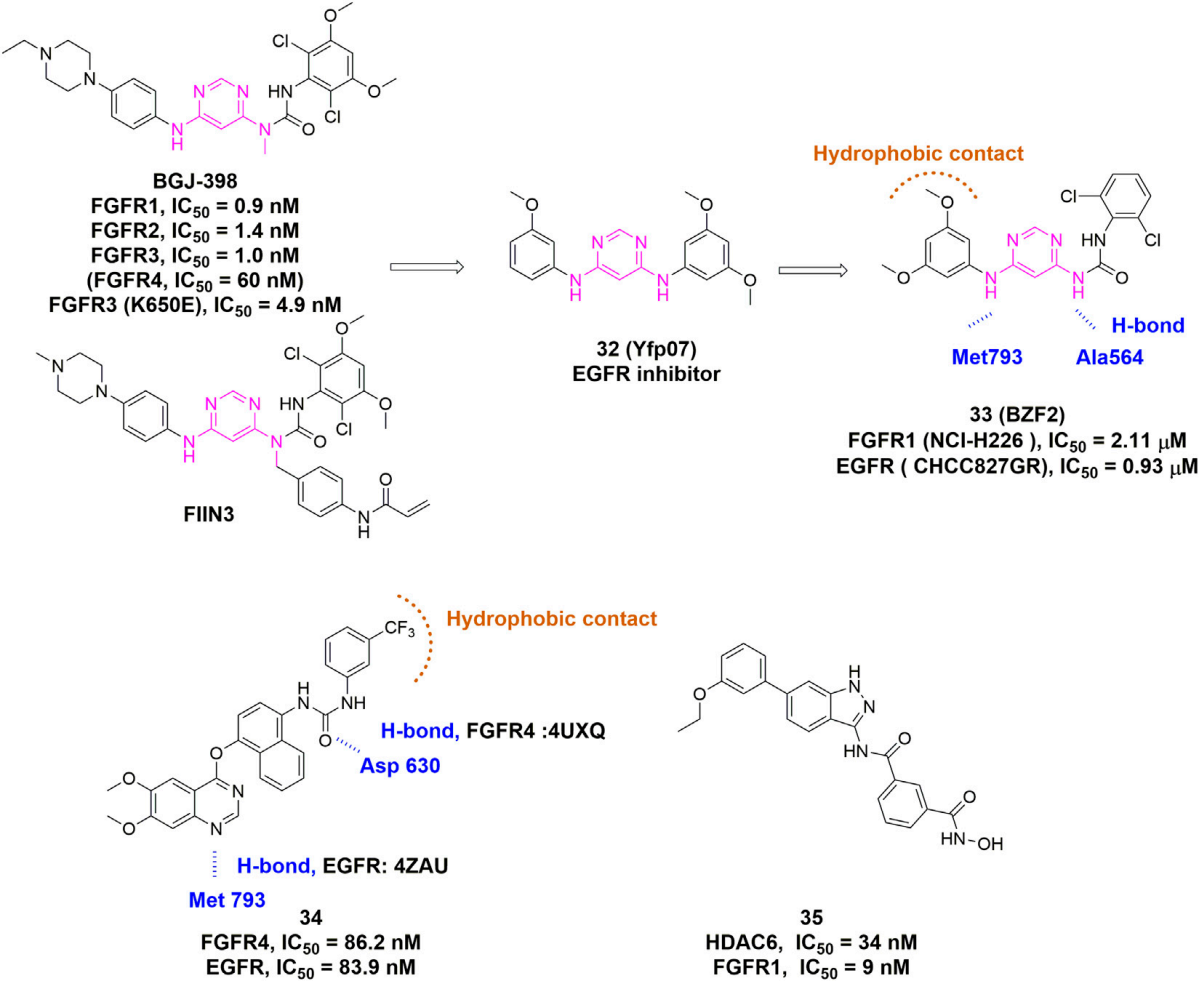
Discovery of representative dual/multi-target inhibitors. H-bonds are outlined as blue hashed lines. Hydrophobic region is indicated by brown arc.

Recently, several publications have paved the way for the rational design of dual/multi-target FGFR inhibitors. Chen *et al.* applied SVM machine learning algorithm to establish QSAR models for FGFR4 and EGFR, which led to the identification of **Cpd 34** as a potent inhibitor of FGFR and EGFR with similar IC_50_ values but distinct binding modes (Figure 14) (Chen et al., 2020). Xie et al*.* developed a series of 4,6-pyrimidinediamine derivatives through incorporation of key scaffolds from FGFR inhibitors (FIIN3 and infigratinib) and EGFR inhibitors (Figure 14, **Cpd 32**) (Sacks et al., 2018). The most promising compound, **BZF2 (** Figure 14, **Cpd 33)**, potently inhibited cell proliferation and cell migration, and induced apoptosis in NSCLC cell lines with FGF2-FGFR1 autocrine loop. Moreover, it exhibited outstanding *in vivo* anti-tumor activity. Apart from FGFR/EGFR dual inhibitors, the FGFR/HDAC dual inhibitors were also reported by Liu et al. (2018). The 1-H-indazol-3-amine-derived FGFR/HDAC dual inhibitor **(** Figure 14, **Cpd 35)** exhibited HDAC6 and FGFR1 dual inhibitions with IC_50_ values of 34 and 9 nM, respectively.

### 5.3 FGFR Degraders

**FIGURE 15:**
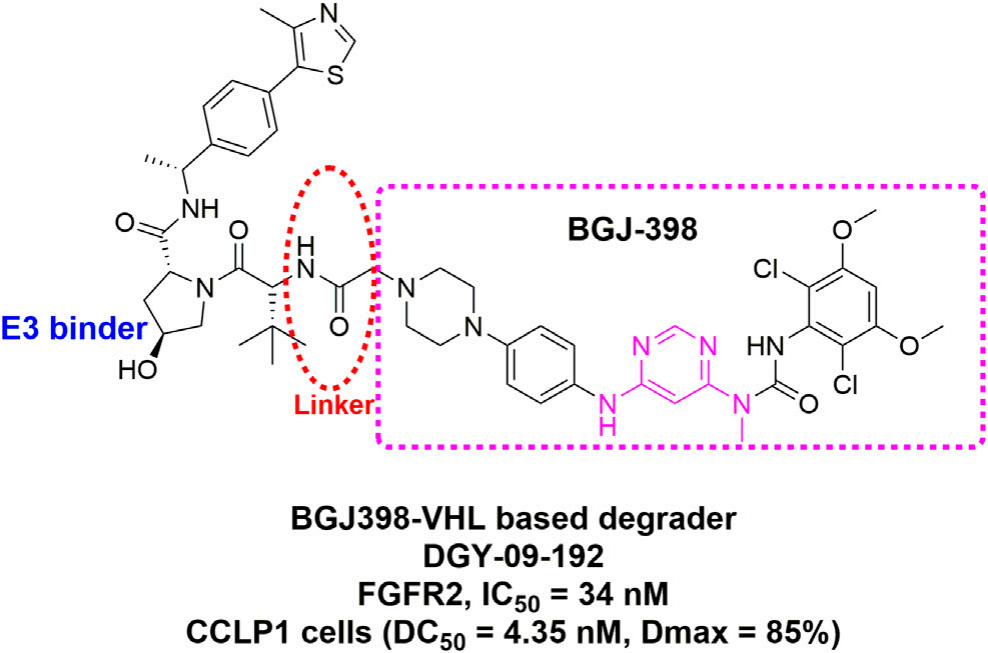
Chemical structure of potent FGFR 1/2 degrader DGY-09–192.

Proteolysis targeting chimera (PROTAC) was first reported by the Crews and Deshaies laboratories in 2001 (Hu et al., 2020; Pettersson et al., 2019). Rather than acting as conventional inhibitors, PROTACs induce selective intracellular proteolysis of target proteins. This novel strategy is likely to circumvent the common disadvantages of traditional occupancy-driven inhibitors such as the toxicity due to off-target and drug resistance caused by compensatory feedback activation of alternative kinases (Paiva et al., 2019). Several kinase targets employing the PROTAC strategy have been explored, including EGFR, HER2, c-Met, ALK, Akt, CK2, ERK1/2, FLT3, PI3K, BTK, RIPK2, and BCR-ABL, most of which are cytosol- or nuclei-located proteins. As for membrane-associated tyrosine kinase receptors like EGFR, Burslem et al. (2018) conjugated a kinase inhibitor Lapatinib to a VHL ligand for degradation of EGFR, HER2, and c-Met. Interestingly, the PROTAC mediated the internalization of EGFR and sorted to lysosomal degradation, although the RTKs usually prefer to be internalized into a recycling endosome (Zou et al., 2019). Du et al. (2021) recently reported a bivalent degrader DGY-09-192, which coupled pan-FGFR inhibitor BGJ-398 to a CRL2^VHL^ E3 ligase (Figure 15). Surprisingly, DGY-09-192 preferentially induced FGFR1 and FGFR2 degradation while largely sparing FGFR3 and FGFR4. Despite multiple concerns regarding cellular permeability, the feasibility of scalable synthesis, and so on (Goracci et al., 2020; Liu et al., 2020), these pioneering studies have demonstrated that the PROTAC approach has a great potential to expand the arsenal against a variety of FGFRs-altered cancers.

## 6 Concluding Remarks

As a type of membrane receptor, FGFR plays a critical role in cell signal transduction and mediates diverse cellular events and processes through a ligand-dependent characteristic. Genetic amplification, mutation, and/or fusion of *FGFR* occurring frequently in various kinds of cancers, can over-activate downstream signaling pathways and cause excessive common oncogenic inducements, such as cell proliferation, inadequate cell apoptosis and cell transformation.

Due to the increasing incidence of abnormal FGF/FGFR signaling axis in various malignancy, such as breast cancers, lung cancers and bladder cancers, FGFRs have been recognized as attractive therapeutic targets. A great number of FGFR inhibitors have been developed in the past decades. Erdafitinib, Pemigatinib and Infigratinib were approved by FDA in 2019, 2020 and 2021, respectively, to treat limited subsets of bladder cancer and cholangiocarcinoma patients with corresponding FGFR alterations, while dozens of other inhibitors are racing in preclinical and clinical development.

TKIs make up a major portion of FGFR-targeting small molecules. The first-generation FGFR inhibitors are general TKIs with a wide spectrum of inhibitory effects for multiple kinases. In contrast, second-generation FGFR inhibitors have improved selectivity, potency and lowered drug resistance, as a result of delicate structure-based design focusing on either optimizing non-covalent interactions with ATP-binding site or the use of covalent warheads to modify critical Cys residues.

The FGFR2 selective inhibitors generated by *de novo* design hold great promise for targeting specific members of FGFR1-3. These inhibitors may possess higher safety, as pan-FGFR inhibitors often display “FGFR1-specific” toxicity that leads to adverse side effects which are presumably originated from abnormal signaling of FGF23 (Chae et al., 2017).

Macrocyclization may serve as another potential strategy for novel FGFR inhibitors. Generally, macrocyclic molecules offer superior binding affinity with targets bearing large and featureless pockets. As seen in other kinase inhibitors, macrocyclic inhibitors also exhibit improved cell permeability, plasma stability and oral absorption when compared to traditional small molecules (Engelhardt et al., 2019; Begnini et al., 2021).

Although there is no allosteric TKI reported for FGFR, this strategy was applied to overcome the drug-resistant EGFR T790M mutant. Jia et al. (2016) described the rational discovery of EAI045, a fourth-generation EGFR inhibitor that targeted an allosteric pocket of the EGFR mutant but not the wild-type kinase. Given the structural similarity between EGFR and FGFR, this finding could give a hint about the design of allosteric TKIs for the latter.

Compared with conventional CADD, it is believed that artificial intelligence (AI) can expand chemical space in a more comprehensive way. Zhavoronkov et al. (2019) established the first deep-learning-based *de novo* design method (GENTRL) to discover inhibitors for a receptor tyrosine kinase called DDR1. In addition, the aforementioned work regarding FGFR4/EGFR dual inhibitor by Chen et al. (2020) also demonstrated the great potential of AI for the discovery of novel FGFR inhibitors with different modes of action.

Small molecules other than TKIs, such as extracellular domain binders or natural products, is another potential source of novel modalities. Many of these molecules show selectivity towards certain subtype of FGFR, probably because the extracellular domains of FGFR1-4 are structurally more distinct than kinase domains. The rapidly emerging resistance to current TKIs may also be overcome by these novel types of molecules. Additionally, it seems unnecessary to impose harsh criteria for cell permeability of these molecules as they typically function outside cells.

Combination therapy and dual/multi-target inhibitor are conceptually similar, while the latter apparently has more advantages since it circumvents any drug-drug interactions. While the rational design of dual inhibitors of FGFR/EGFR, FGFR/VEGFR, and FGFR/HDAC may inspire rapid discovery of more inhibitors simultaneously acting on FGFR and another target, identification of more genes that can robustly cause synthetic lethality with *FGFR* is the central problem and requires extensive in-depth research.

The thriving techniques of targeted protein degradation including PROTAC, molecular glue, as well as other TACs (AUTAC, LYTAC, ATTEC, etc.), have demonstrated broad applicability. PROTACs for RTKs including EGFR and FGFR, have been recently reported, all of which utilized current kinase inhibitors as the RTK binders. It can be envisaged that small molecules occupying a pocket of the kinase beyond ATP-binding site are especially suitable for designing new PROTACs. Such PROTACs are supposed to improve the selectivity and offer solutions to combat drug resistance.

This review encompassed most of the existing FGFR inhibitors and elaborated important structures from a medicinal chemistry perspective. We anticipate that more and more tailor-made novel small molecules of different types and modalities will be developed to improve future targeted therapy with higher efficacy and lower toxicity.
