# Supplementary material for: Signaling Pathway and Small-Molecule Drug Discovery of FGFR: A Comprehensive Review
Source: Front Chem. 2022 Apr 14;10:860985. doi: 10.3389/fchem.2022.860985 (PMC9046545; doi:10.3389/fchem.2022.860985)
Supplement: Supplementary file 1 [file DataSheet1.pdf]

Appendix.

Signaling pathway and small-molecule drug discovery of FGFR: A comprehensive review

Jia Zheng[1], Wei Zhang[1], Linfeng Li[1], Yi He[1], Yue Wei[1], Yongjun Dang[1], Shenyou Nie[1,2*], Zufeng Guo[1,2*]

[1]Center for Novel Target and Therapeutic Intervention, Institute of Life Sciences, Chongqing Medical University, Chongqing, China.

***Correspondence**:
Shenyou Nie, nieshenyou@cqmu.edu.cn;
Zufeng Guo, zfguo@cqmu.edu.cn

Appendix A. Summary of FGFR inhibitors						
**Name**	**Target**	**Indication**	**Clinical stage**	**Company or Sponsor**	**IC_{50} ***	**References**
Ponatinib (AP24534)	Abl, PDGFRα, VEGFR, FGFR1	Lung cancer<br>Acute and chronic leukemia	Phase 2 (NCT01935336)<br>Phase 1/2 (NCT02398825; NCT02428543)	Ariad Pharmaceuticals	FGFR1, IC_{50}=2.2 nM	(O'Hare et al., 2009)
Nintedanib (BIBF1120)	VEGFR, FGFR, PDGFR	Advanced pancreatic cancer	Phase 1/2 (NCT02902484)	Boehringer Ingelheim	FGFR1/2/3, IC_{50}=69/37/108 nM	(Hilberg et al., 2008)
Dovitinib (TKI25)	c-Kit,VEGFR, PGFR, FLT, FGFR1/2/3	Hormone refractory prostate cancer	Phase 2 (NCT01741116)	Novartis Pharmaceuticals	FGFR1/3, IC_{50}=8/9 nM	(Trudel et al., 2005)
S49076	MET, AXL, FGFR1/2/3	Advanced solid tumors	*NA**	Oncology Research and Development Unit, Institut de Recherches Servier	FGFR1/2/3,IC_{50}=18 nM	(Burbridge et al., 2013)
Orantinib (TSU-68, SU6668)	VEGFR-2, FGFR, PGFGR	Hepatocellular carcinoma	Phase 3 (NCT01465464)	Taiho Pharmaceutical Co., Ltd.	FGFR1, K_i =1.2 µM	(Laird et al., 2000)
Lenvatinib (E7080)	RET, VEGFR, KDR, FLT, FGFR	Relapsed or refractory solid tumors	Phase 2 (NCT04447755)	Eisai Inc.<br>Merck Sharp & Dohme Corp.	FGFR1, IC_{50}=46 nM	(Matsui et al., 2008)
Brivanib Alaninate (BMS-582664)	VEGFR, Flk1, VEGFR, FGFR	Cervical cancer	Phase 2 NCT04395612	Bristol-Myers Squibb	FGFR1, IC_{50}=148 nM	(Bhide et al., 2006)
Brivanib (BMS-540215)	VEGFR, FGFR 1	Cervical cancer	Phase 3 (NCT04395612)	Bristol-Myers Squibb	FGFR1, IC_{50}=148 nM	(Bhide et al., 2006)

Lucitanib (E3810)	VEGFR, FGFR2, PDGFR	Solid tumors	Phase 1/2 (NCT01283945)	Eisai Inc.	FGFR1/2, IC_{50}=17.5/ 82.5 nM	(Bello et al., 2011)
SKLB 610	VEGFR2, FGFR2, PDGFR	Non-small cell lung cancer	Preclinical study	State Key Laboratory of Biotherapy and Cancer Center, West China Hospital, West China Medical School, Sichuan University	Inhibition rate (10 μM) = 65%	(Cao et al., 2011)
SSR128129E	FGFR	Behcet's disease	Phase 3 (NCT04528082)	Amgen	FGFR, IC_{50}=1.9 μM	(Bono et al., 2013)
Sulfatinib (HMPL-012)	VEGFR1, VEGFR2, VEGFR3, FGFR1, FMS	Tumor	Early Phase1 (NCT03627520)	Hutchison Medipharma Limited	FGFR1, IC_{50}=15 nM	(J. M. Xu et al., 2017)
Pazopanib (GW786034)	VEGFR1, VEGFR2/3, PDGFR，FGFR, c-Kit, c-fms	Medical treatment for metastatic renal cell carcinoma	NCT04659343	Frede Donskov|Odense University Hospital|Herlev Hospital|Rigshospitalet Denmark|University of Aarhus	FGFR, IC_{50}=74 nM	(Harris et al., 2008)
Danusertib (PHA-739358)	AuroraA/B/C, Abl，TrkA, c-RET, FGFR1	Multiple myeloma	Phase 2 (NCT00872300)	Nerviano Medical Sciences	FGFR1, IC_{50}=47 nM	(Carpinelli et al., 2007)
ENMD-2076	AuroraA, FLT3, RET, SRC, NTRK1, FMS, VEGFR2, FGFR, PDGFRα	Ovarian clear cell carcinoma	Phase 2 (NCT01914510)	University Health Network, Toronto	FGFR1, IC_{50} =92.7 nM	(Fletcher et al., 2011)
MK-2461	c-Met, Ron, FLT1/3/4, FGFR1/2/3, PDGFRβ, KDR	c-Met and FGFR2-driven tumors	Phase 1 (NCT00518739)	Merck Sharp & Dohme Corp.	FGFR1/2/3, IC_{50}=65/39/50 nM	(Pan et al., 2010)

Tyrphostin AG 1296	PDGFR, FGFR, c-Kit	Cancers	Preclinical study	*NA*	FGFR (Swiss 3T3), IC_{50}= 12.3 μM	(Kovalenko et al., 1994)
ON123300	PDGFRβ, FGFR, RET, Fyn	Solid tumors, adult (Carpinelli et al., 2007)	Phase 1 (NCT04739293)	Onconova Therapeutics, Inc.	FGFR1, IC_{50}=26 nM	(X. Zhang et al., 2014)
SU 5402	VEGFR2, FGFR1, PDGFRβ	Cancers	Preclinical study	Department of Pharmacology, New York University Medical Center	FGFR1, IC_{50}=30 nM	(Sun et al., 1999)
Erdafitinib (JNJ42756493)	FGFR1-4	Locally advanced or metastatic bladder cancer, etc.	Approved in 2019	Janssen Research & Development, LLC	FGFR1/2/3/4, IC_{50}=1.2/2.5/3.0/5.7 nM	(Perera et al., 2017)
ASP-5878	FGFR1-4	Solid tumors	Phase 1 (NCT02038673)	Astellas Pharma Inc	FGFR1/2/3/4, IC_{50}= 0.47/0.6/0.74/0.74 nM	(Futami et al., 2017)
Derazantinib (ARQ-087)	FGFR1-4	Advanced intrahepatic cholangiocarcinoma with FGFR2 Gene amplifications/ mutations/ fusions	Phase 2 (NCT03230318)	Basilea Pharmaceutica	FGFR1/2/3/4, IC_{50}=4.5/1.8/4.5/34 nM	(Hall et al., 2016)
Rogaratinib (BAY1163877)	FGFR1-4	Neoplasms	Phase 1 (NCT03788603)	Bayer	FGFR1/2/3/4, IC_{50}=1.8/<1/9.2/1.2 nM	(Grunewald et al., 2019)
FIIN-1	FGFR1-4	Drug-resistant tumors	Preclinical study	Harvard Medical School	FGFR1/2/3/4, IC_{50}= 9.2/6.2/11.9/182 nM	(Zhou et al., 2010)
FIIN-2	FGFR1-4	Drug-resistant tumors	Preclinical study	Harvard Medical School	FGFR1/2/3/4, IC_{50}= 3.09/4.3/27/45.3 nM	(Tan et al., 2014)
FIIN-3	FGFR1-4	Drug-resistant tumors	Preclinical study	Harvard Medical School	FGFR1/2/3/4, IC_{50}= 13.1/21/31.4/35.3 nM	(Tan et al., 2014)
LY2874455	FGFR1-4	Advanced cancer	Phase 1 (NCT01212107)	Eli Lilly	FGFR1/2/3/4, IC_{50}=2.8/2.6/6.4/6 nM	(G. Zhao et al., 2011)

PRN-1371	FGFR1-4	Metastatic urothelial carcinoma & renal pelvis & ureter	Phase 1 (NCT02608125)	Principia Biopharma, a Sanofi Company	FGFR1/2/3/4, IC_{50}= 0.6/1.3/4.1/19.3 nM	(Brameld et al., 2017)
Futibatinib (TAS-120)	FGFR1-4	Metastatic breast cancer with FGFR2 amplification	Phase 2 (NCT04024436)	Taiho Oncology, Inc.	FGFR1/2/3/4, IC_{50}=1.8/1.4/1.6/3.7 nM	(Sootome et al., 2020)
ICP-192	FGFR1-4	Urothelial carcinoma, cholangiocarcinoma	Phase 1/2 (NCT04565275)	Beijing InnoCare Pharma Tech Co., Ltd.	*NA*	https://clinicaltrials.gov/ct2/show/NCT04565275
Pemigatinib (INCB054828)	FGFR1/2/3	Unresectable or metastatic cholangiocarcinoma	Approved in 2020	Incyte Corporation	FGFR1/2/3/4, IC_{50}=0.4/0.5/1.2/30 nM	(Roskoski, 2020)
Infigratinib (BGJ398)	FGFR1/2/3	Advanced or metastatic cholangiocarcinoma	Approved in 2021	BridgeBio Pharma	FGFR1/2/3/4, IC_{50} =0.9/1.4/1.0/60 nM FGFR3 (K650E), IC_{50} =4.9 nM	(Guagnano et al., 2011)
AZD4547	FGFR1/2/3	ER+ breast cancer	Phase 2/3 (NCT01202591)	AstraZeneca	FGFR1/2/3, IC_{50}=0.2/2.5/8 nM	(Gavine et al., 2012)
CPL304110	FGFR1/2/3	Advanced solid malignancies	Phase 1 (NCT04149691)	Celon Pharma S.A.	FGFR1/2/3/4, IC_{50}= 0.75/0.50/3.05/87.9 nM	(Yamani et al., 2021)
Zoligratinib (Debio1347)	FGFR1/2/3	Solid malignancies	Phase 1 (NCT01948297)	Debiopharm International SA	FGFR1/2/3/4, IC_{50}= 9.3/7.622/290 nM	(Nakanishi et al., 2014)

E7090	FGFR1/2/3	Cholangiocarcinoma	Phase 1 (NCT04565574)<br>Phase 2 (NCT04238715)	Eisai Co., Ltd.	FGFR1/2/3/4, IC_{50}= 0.71/0.50/1.2/120 nM<br>FGFR3 (K650E), IC_{50}=3.1 nM<br>FGFR3 (K650M), IC_{50} =16 nM	(Watanabe Miyano et al., 2016)
3D185	FGFR1/2/3	Advanced solid tumors, Cholangiocarcinoma	Phase 1 (NCT04221204)<br>Phase 2 (NCT05039892)	3D Medicines Co., Ltd.	FGFR1/2/3, IC_{50} = 0.5/1.3/3.6 nM	(Peng et al., 2019)
BLU9931	FGFR 4	Hepatocellular carcinoma	Preclinical study	Blueprint Medicines Corporation	FGFR4, IC_{50} =3 nM	(Hagel et al., 2015)
Fisogatinib (BLU554)	FGFR 4	Advanced solid tumors, Hepatocellular carcinoma (HCC)	Phase 1b/2 (NCT04194801)	Blueprint Medicines Corporation	FGFR4, IC_{50}=5 nM (FGFR1-3, IC_{50}=624-2203 nM)	Blueprint Medicines. 50th th international liver congress. 2015
FGF401	FGFR 4	Solid malignancies, Hepatocellular carcinoma (HCC)	Phase 1/2 (NCT02325739)	Novartis Pharmaceuticals	FGFR4, IC_{50}=1.1 nM	(Fairhurst et al., 2020)
H3B-6527	FGFR 4	Advanced hepatocellular carcinoma	Phase 1 (NCT02834780)	H3 Biomedicine, Cambridge, Massachusetts	FGFR4, IC_{50} < 1.2 nM	(Joshi et al., 2017)
ICP-105	FGFR 4	Advanced Solid Malignancies	Phase 1 (NCT03642834)	Beijing InnoCare Pharma Tech Co., Ltd.	*NA*	https://www.clinicaltrials.gov/ct2/show/NCT0364283
EVER4010001	FGFR 4	Advanced Solid Tumors	Phase 1/2 (NCT04699643)	Chinese PLA General Hospital	Combination With PD-1 Inhibitor Pembrolizumab	https://www.clinicaltrials.gov/ct2/show/NCT04699643

Vofatamab (B-701)	FGFR 3 (Anti-FGFR3 monoclonal antibody)	Locally advanced or metastatic urothelial cell Carcinoma	Phase 1/2 (NCT02401542)	Rainier Therapeutics	Combination With Pembrolizumab	https://www.clinicaltrials.gov/ct2/show/NCT03123055
BO-264	FGFR3-TACC3	Specifically blocking the function of FGFR3-TACC3 fusion protein	Preclinical study	Department of Molecular Biology and Genetics, Faculty of Science, Bilkent University, Ankara, Turkey.	*NA*	(Akbulut et al., 2020)
Masitinib (AB1010)	FGFR 3	Multiple myeloma	Preclinical study	AB Science	*NA*	(Dubreuil et al., 2009)
RLY4008	FGFR 2	Solid tumor	Phase 1 (NCT04526106)	Relay Therapeutics, Inc.	*NA*	https://clinicaltrials.gov/ct2/show/NCT04526106
Alofanib (RPT385)	FGFR 2	Ovarian cancer	Preclinical study	Ruspharmtech LLC	allosteric FGFR2 inhibitor, IC_{50} < 10 nM	(Tyulyandina et al., 2017)
NSC12	FGFs (Small-Molecule extracellular FGF Trap)	Cancer	Preclinical study	Department of Molecular and Translational Medicine, University of Brescia	FGF2, IC_{50} =30 nM	(Ronca et al., 2015)
Rosmarinic acid	FGF2/FGFR-D2 complex	FGF2-mediated drug resistance	Preclinical study	RagonaIstituto di Scienze e Tecnologie Chimiche (SCITEC), CNR Institution	IC_{50} =17.7 ±2.1 μM	(Pagano et al., 2021)
PD-166866	FGFR1	Small cell lung cancer cell	Preclinical study	Warner-Lambert Company, Ann Arbor, Michigan, USA.	FGFR1, IC_{50} = 52.4 nM	(Risuleo et al., 2009)

SU-4984	FGFR1	Malignant glioma and cancers	Preclinical study	State Key Laboratory of Biotherapy and Cancer Center, West China Hospital, West China Medical School, Sichuan University	FGFR1, IC_{50}=20-40 μM	(Mohammadi et al., 1997)
Gambogenic acid	FGFR	Erlotinib-resistant non-small-cell lung cancer	Preclinical study	Shanghai University of Traditional Chinese Medicine	IC_{50} of H1650 cells/HCC827 cells/ HCC827ER cells = 0.909/1.328/1.51 μM	(L. Xu et al., 2018)
Ferulic Acid	FGFR1/2	Melanoma	Preclinical study	Zhejiang Provincial People's Hospital	FGFR1/2, IC_{50}=3.78/12.5 μM	(G. W. Yang et al., 2015)
Cediranib (AZD2171)	VEGFR, FGFR	Advanced solid tumor, FGFR positive lung cancer	Phase 1 (NCT00979134) Phase 2/3 (NCT02965378)	AstraZeneca	FGFR1, IC_{50}= 26 nM	(Wedge et al., 2005)
AT9283	FGFR1/2/3	Non-Hodgkins lymphoma Unspecified adult solid tumor	Phase 1 (NCT00443976)	Astex Pharmaceuticals, Inc.	FGFR1/2/3, IC_{50}= 10-30/1-10/1-30nM FGFR1 (V561M), IC_{50}=10-30 nM FGFR2 (N549H), IC_{50}=10-30 nM	(Howard et al., 2009)
BZF-2	EGFR, FGFR	EGFR/FGFR1-positive NSCLC	Preclinical study	School of Pharmaceutical Science, Wenzhou Medical University,	NCI-H226, IC_{50}= 2.11 μM HCC827GR, IC_{50} = 0.93 μM	(Xie et al., 2020)

Ki23057	VEGFR, FGFR	Drug-resistant gastric cancer	Preclinical study	Kirin Brewery Co.,Ltd. (Gunma, Japan)	FGFR1/2, IC_{50} = 89/91 nM VEGFR2, IC_{50}= 38 nM	(Qiu et al., 2011)
MPT0L145	PI3K, FGFR	Bladder cancer	Preclinical study	Department of Pharmacology, School of Medicine, College of Medicine, Taipei Medical University	FGFR-dependent/independent, IC_{50}=1.83/6.74 μM	(C. H. Chen et al., 2016)
ODM-203	VEGFR, FGFR	Solid tumors	Phase 1 (NCT02264418)	Orion Corporation Orion Pharma, Finland	FGFR1/2/3/4, IC_{50} = 11/16/6/35 nM VEGFR1/2/3, IC_{50} = 26 /9/5 nM	(Holmström et al., 2019)
SOMCL-085	VEGFR, FGFR	FGFR-driven cancers	Preclinical study	Shanghai Institute of Materia Medica	FGFR1/2/3, IC_{50}= 1.8/1.9 /6.9 nM	(Jiang et al., 2018)
SOMCL-286	VEGFR, FGFR	FGFR1/2 and VEGFR2 addictive cancer	Preclinical study	Shanghai Institute of Materia Medica	FGFR1/2/3, IC_{50}= 1.0/4.5/2.9 nM,	(M. Wei et al., 2018)
PD173074	VEGFR, FGFR	Non-small cell lung cancer cell	Preclinical study	Department of Pharmacology, New York University Medical Center	FGFR1, IC_{50} =1-5 nM VEGFR2, IC_{50} =100-200 nM	(Mohammadi et al., 1998)

FGF, fibroblast growth factor; FGFR, fibroblast growth factor receptor; VEGFR, vascular endothelial growth factor receptor; PI3K, phosphoinositide 3-kinase; EGFR, epidermal growth factor receptor; TACC, transforming acidic coiled-coil containing protein; PDGFR, platelet-derived growth factor receptor; RET, rearranged during transfection; Fyn, fyn proto-oncogene, src family tyrosine kinase; AXL, axl receptor tyrosine kinase; Flk, fetal liver kinase; FMS, colony-stimulating factor 1 receptor; Abl, Abl tyrosine kinase; AuroraA, aurora kinase A; SRC, src proto-oncogene, non-receptor tyrosine kinase; NTRK1, neurotrophic receptor tyrosine kinase; Ron, macrophage stimulating 1 receptor; KDR, kinase insert domain receptor; Flt, fms related receptor tyrosine kinase; Trk neurotrophic receptor tyrosine kinase; Kit, kit proto-oncogene receptor tyrosine kinase. *IC_{50}: The reference of IC_{50} have been cited in the table. **NA*: Not available.

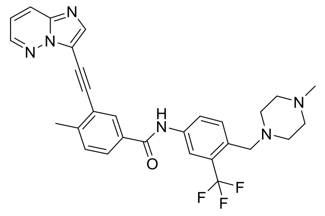

Ponatinib, AP24534
Abl, IC_{50}=0.37 nM
PDGFR, IC_{50}=1.1 nM
VEGFR2, IC_{50}=1.5 nM
FGFR1, IC_{50}=2.2 nM
Src, IC_{50}=5.4 nM

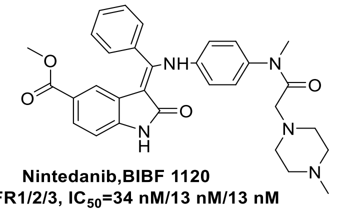

Nintedanib,BIBF 1120
VEGFR1/2/3, IC_{50}=34 nM/13 nM/13 nM
FGFR1/2/3, IC_{50}= 69 nM/37 nM/108 nM
PDGFRα/β, IC_{50}=59 nM/65 nM

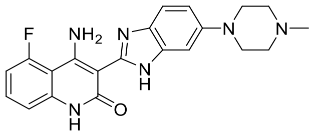

Dovitinib, TKI258
FLT3/c-Kit, IC_{50}=1 nM/2 nM
FGFR1/3 and VEGFR1-4, IC_{50}= 8-13 nM

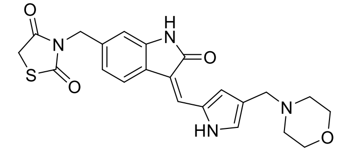

S49076
FGFR1/2/3,IC_{50}<20 nM

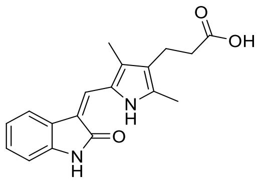

Orantinib (TSU-68, SU6668)
FGFR1, Ki = 1.2 μM

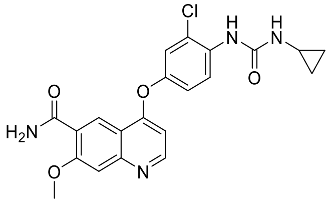

Lenvatinib, E7080
VEGFR2(KDR)/VEGFR3(Flt-4)
IC_{50}=4 nM/5.2 nM
FGFR1-4,PDGFR,Kit (c-Kit),RET (c-RET)

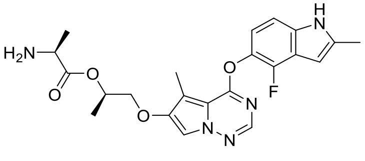

BMS-582664
FGFR1, IC_{50}= 148 nM

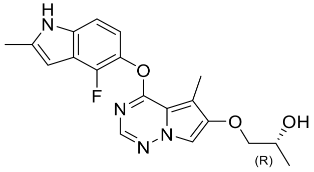

BMS-540215
FGFR1, IC_{50}= 148 nM

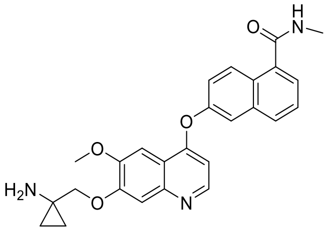

Lucitanib, E3810
FGFR1, IC_{50}= 17.5 nM
FGFR2, IC_{50}= 82.5 nM

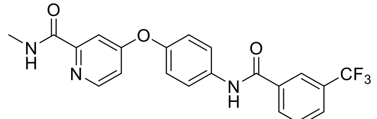

SKLB 610
Inhibiting FGFR2 at a rate of 65%, at the concentration of 10 μM

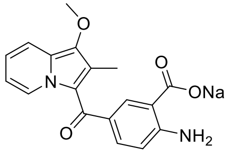

SSR128129E
FGFR, IC_{50}=1.9μM

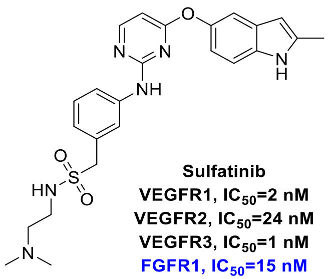

Sulfatinib
VEGFR1, IC_{50}=2 nM
VEGFR2, IC_{50}=24 nM
VEGFR3, IC_{50}=1 nM
FGFR1, IC_{50}=15 nM
CSF1R, IC_{50}=4 nM

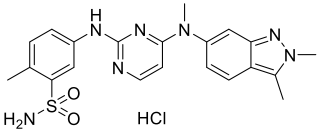

Pazopanib HCl (GW786034 HCl)
VEGFR1/2/3,
IC_{50}=10 nM/30 nM/47 nM
PDGFR,FGFR,c-Kit,c-fms
IC_{50}=84 nM, 74 nM,140 nM,146 nM

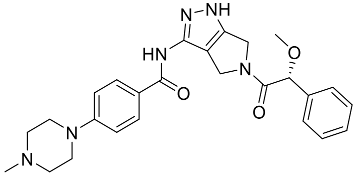

Danusertib (PHA-739358)
Aurora A/B/C, IC_{50}=13 nM/79 nM/61 nM
Abl,TrkA,c-RET, FGFR1,Lck,
VEGFR2/3,c-Kit,CDK2, weak activity

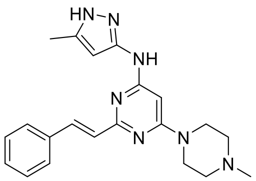

ENMD-2076
Aurora A, IC_{50}=14 nM,
Flt3, IC_{50}=1.86 nM,
RET,SRC,NTRK1/TRKA,
CSF1R/FMS,VEGFR2/KDR,
FGFR, PDGFRα, weak activity

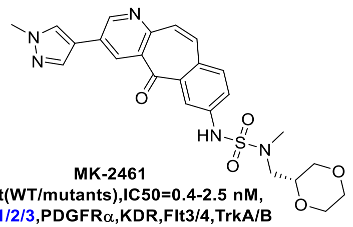

MK-2461
c-Met(WT/mutants),IC50=0.4-2.5 nM,
FGFR1/2/3,PDGFRα,KDR,Flt3/4,TrkA/B

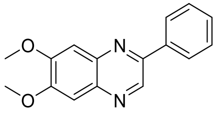

Tyrphostin, (AG 1296)
PDGFR,IC_{50}=0.3-0.5 μM
FGFR, IC50 =12.3 μM (Swiss 3T3)
c-Kit,IC50 =1.8 μM (Swiss 3T3)

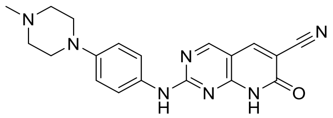

ON123300
CDK4,Ark5/NUAK1,PDGFRα,FGFR1,
RET (c-RET),Fyn,
IC_{50}=3.9 nM,5 nM, 26 nM, 26 nM, 9.2 nM, 11nM

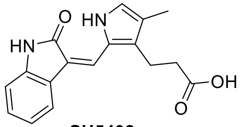

SU5402
VEGFR2,FGFR1,PDGF-Rβ
IC_{50}=20 nM,30 nM,510 nM

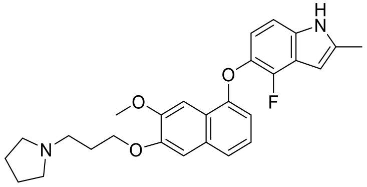

Cediranib, AZD-2171
FGFR1, IC_{50}= 26 nM

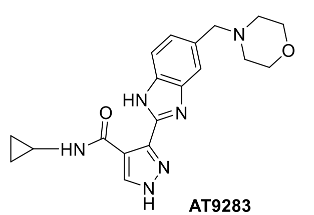

AT9283
FGFR2, IC_{50}= 1-10 nM
FGFR1, IC_{50}=10 nM-30 nM
FGFR1 (V561M), IC_{50}=10 nM-30 nM
FGFR2 (N549H), IC_{50}=10 nM-30 nM
FGFR3, IC_{50}=10 nM-30 nM

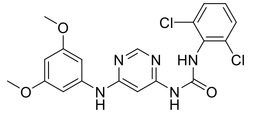

33 (BZF2)
FGFR1 (NCI-H226), IC_{50} = 2.11 μM
EGFR (CHCC827GR), IC_{50} = 0.93 μM

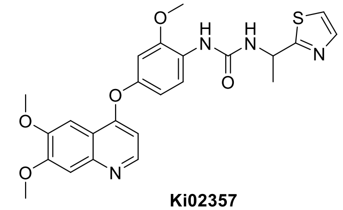

Ki02357
K-samII/FGF-R2, IC_{50} = 91 nM
FGF-R1, IC_{50}= 89 nM
(VEGF-R2, IC_{50}= 38 nM)

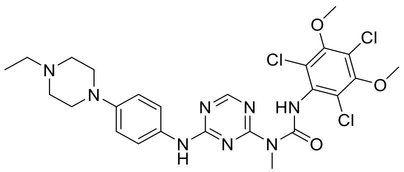

MPT0L-145
IC_{50} =1.83 nM (FGFR-dependent cells)

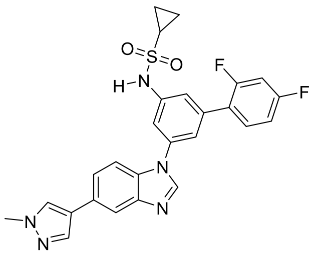

ODM-203
FGFR1, IC_{50} = 11 nM
FGFR2, IC_{50} = 16 nM
FGFR3, IC_{50} = 6 nM
FGFR4, IC_{50} = 35 nM
(VEGFR1, VEGFR2, VEGFR3,
IC_{50} = 26 nM, 9 nM, 5 nM)

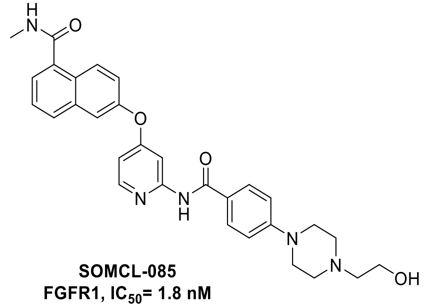

SOMCL-085
FGFR1, IC_{50}= 1.8 nM
FGFR2, IC_{50}= 1.9 nM
FGFR3, IC_{50}= 6.9 nM

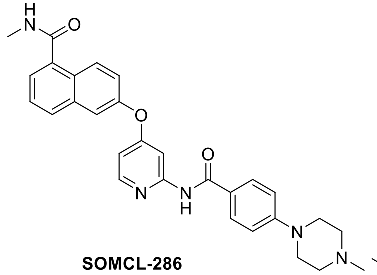

SOMCL-286
FGFR1, IC_{50}= 1.0 nM
FGFR2, IC_{50}= 4.5 nM
FGFR3, IC_{50}= 2.9 nM

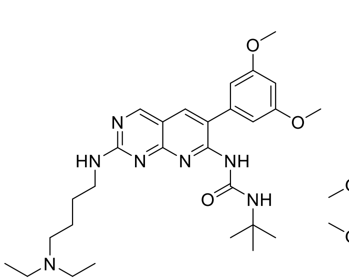

PD173074
FGFR1, IC_{50} = 50 nM
VEGFR2, IC_{50} = 100-200 nM

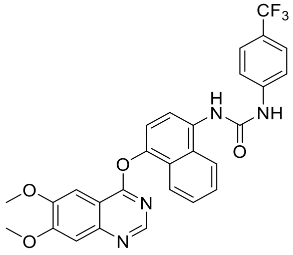

34
dual-target FGFR
FGFR4, IC_{50} = 86.2 nM
EGFR, IC_{50} = 83.9 nM

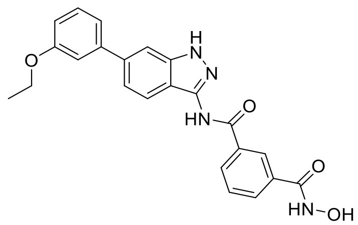

35
1-H-indazol-3-amine derivative
HDAC6, IC_{50} = 34 nM
FGFR1, IC_{50} = 9 nM

Figure S1. Reported multi-target or dual target FGFR inhibitors with targets and IC_{50} values

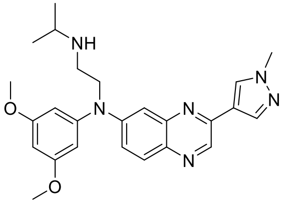

Balversa(Erdafitinib,JNJ42756493)
2019
FGFR1, IC_{50}=1.2 nM
FGFR2, IC_{50}=2.5 nM
FGFR3, IC_{50}=3.0 nM
FGFR 4, IC_{50}= 5.7 nM

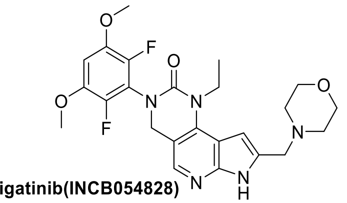

Pemigatinib(INCB054828)
FGFR 1, IC_{50}=0.4 nM
FGFR 2, IC_{50}=0.5 nM
FGFR 3, IC_{50}=1.2 nM
(FGFR 4, IC_{50}=30 nM)

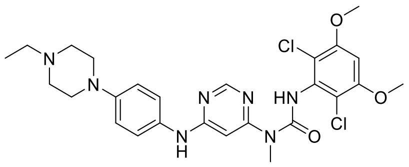

Infigratinib(BGJ398)
FGFR1, IC_{50} =0.9 nM
FGFR2, IC_{50} =1.4 nM
FGFR3, IC_{50} =1.0 nM
(FGFR4, IC_{50} = 60 nM)
FGFR3 (K650E), IC_{50} =4.9 nM

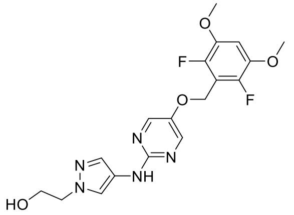

ASP5878
FGFR1, IC_{50}=0.47nM
FGFR2, IC_{50}=0.60nM
FGFR3, IC_{50}=0.74nM
FGFR4, IC_{50}= 0.74nM

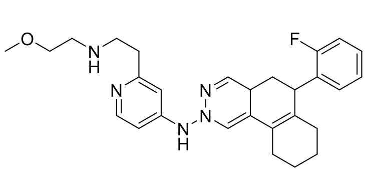

ARQ-087
FGFR1, IC_{50}=4.5 nM
FGFR2, IC_{50}=1.8 nM
FGFR3, IC_{50}=4.5 nM
FGFR4, IC_{50}= 34 nM

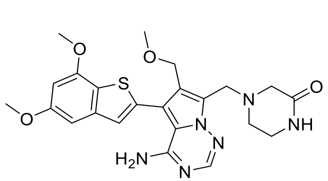

Rogaratinib
(BAY1163877)
FGFR1, IC_{50}=15 nM
FGFR2, IC_{50}=1 nM
FGFR3, IC_{50}=19 nM
FGFR4, IC_{50}= 33 nM

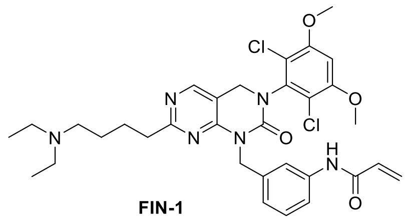

FIN-1
FGFR1, IC_{50}=9.2 nM
FGFR2, IC_{50}=6.2 nM
FGFR3, IC_{50}=11.9 nM
FGFR4, IC_{50}= 182 nM

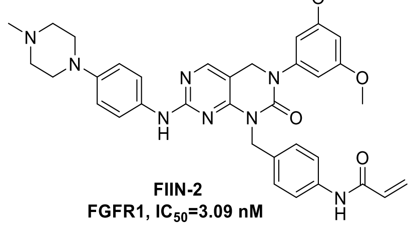

FIIN-2
FGFR1, IC_{50}=3.09 nM
FGFR2, IC_{50}=4.3 nM
FGFR3, IC_{50}=27 nM
FGFR4, IC_{50}=45.3 nM

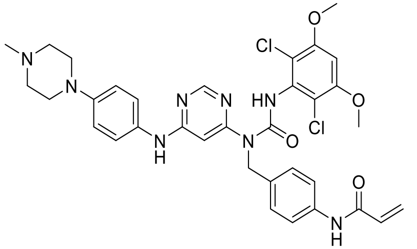

FIIN-3
FGFR1, IC_{50}=13.1 nM
FGFR2, IC_{50}=21 nM
FGFR3, IC_{50}=31.4 nM
FGFR4, IC_{50}= 35.3 nM

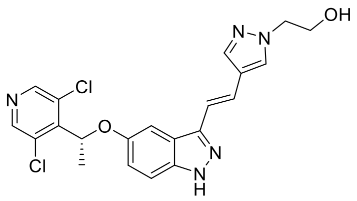

LY287445
FGFR1, IC_{50}=2.8 nM
FGFR2, IC_{50}=2.6 nM
FGFR3, IC_{50}=6.4 nM
FGFR4, IC_{50}=6 nM
VEGFR2, IC_{50}=7 nM

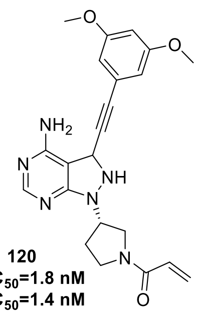

TAS 120
FGFR 1, IC_{50}=1.8 nM
FGFR 2, IC_{50}=1.4 nM
FGFR 3, IC_{50}= 1.6 nM
FGFR 4, IC_{50}=3.7 nM

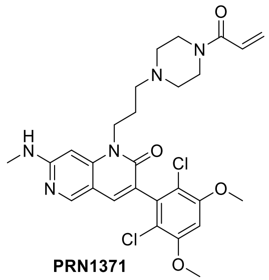

PRN1371
FGFR1, IC_{50}=0.6 nM
FGFR2, IC_{50}=1.3 nM
FGFR3, IC_{50}=4.1nM
FGFR4, IC_{50}=19.3 nM

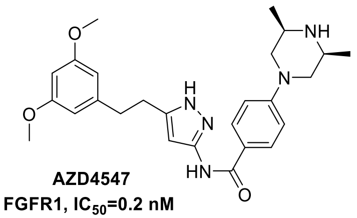

AZD4547
FGFR1, IC_{50}=0.2 nM
FGFR2, IC_{50}=2.5 nM
FGFR3, IC_{50}=8 nM

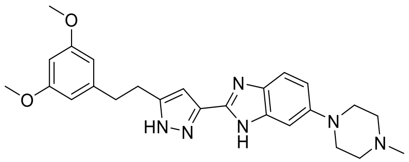

CPL304110
FGFR1, IC_{50} = 0.75 nM
FGFR2, IC_{50} = 0.50 nM
FGFR3, IC_{50} = 3.05 nM
(FGFR4, IC_{50} = 87.9 nM)

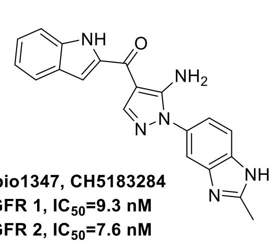

Debio1347, CH5183284
FGFR 1, IC_{50}=9.3 nM
FGFR 2, IC_{50}=7.6 nM
FGFR 3, IC_{50}=22 nM
(FGFR 4, IC_{50}=290 nM)

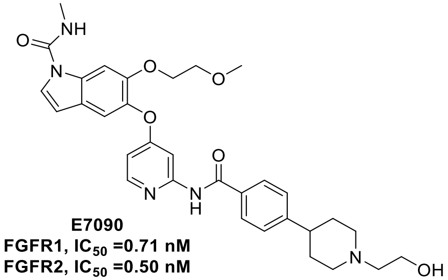

E7090
FGFR1, IC_{50} =0.71 nM
FGFR2, IC_{50} =0.50 nM
FGFR3, IC_{50} =1.2 nM
(FGFR4, IC_{50} = 120 nM)
FGFR3 (K650E), IC_{50} =3.1 nM
FGFR3 (K650M) IC_{50} =16 nM

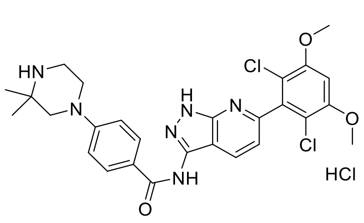

3D185
FGFR1, IC_{50} = 0.5 nM
FGFR2, IC_{50} = 1.3 nM
FGFR3, IC_{50} = 13.6 nM

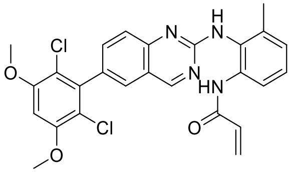

BLU9931
FGFR 4,IC_{50}=3 nM

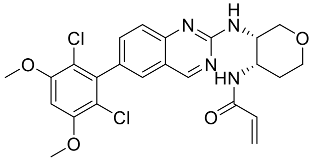

BLU554
FGFR4, IC_{50}=5 nM
(FGFR1-3, IC_{50}: 624-2203 nM)

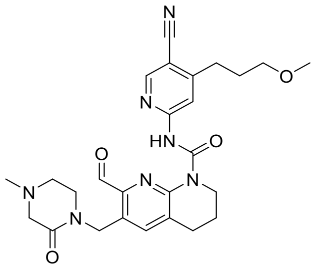

FGF401
FGFR 4,IC$_{50}$=1.1 nM

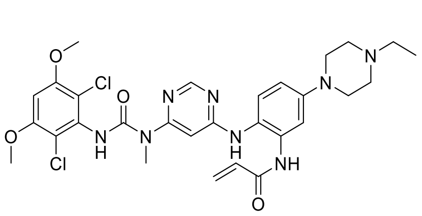

H3B-6527
FGFR 4, IC$_{50}$ < 1.2 nM

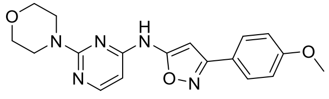

BO-264
IC$_{50}$ = 188 nM
Kd =1.5 nM

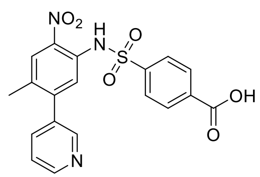

RPT835
FGFR2, IC$_{50}$<10 nM (KATO III)a

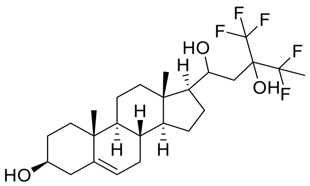

NSC12
FGF2/FGFR, IC$_{50}$ =30 nM

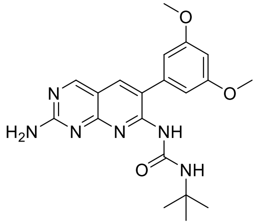

PD-166866
FGFR1, IC$_{50}$ = 52.4 nM

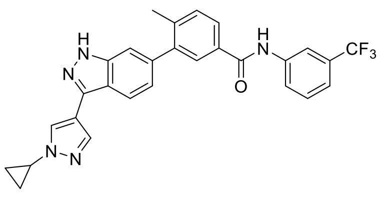

36
3-substituted indazole derivative.

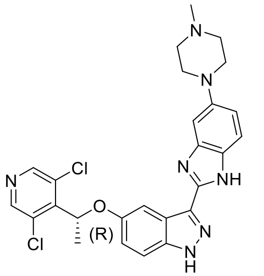

37
3-benzimidazol-5-pyridine alkoxy-1H-indole derivative

Figure S2. Reported Selective FGFR inhibitors and FGFR Traps

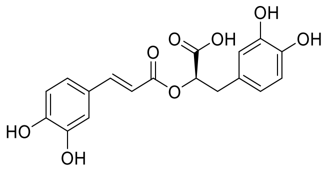

Rosmarinic acid
FGF2/FGFR-D2 complex
IC50 =17.7± 2.1 μM

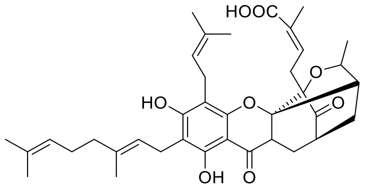

Gambogenic acid
H1650 cells, IC50=0.909 μM
HCC827 cells, IC50=1.328 μM
HCC827ER cells, IC50=1.51 μM

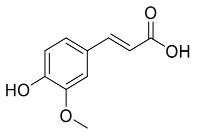

Ferulic Acid
FGFR1, IC_{50}=3.78 μM
FGFR2, IC_{50}=12.5 μM

Figure S3. Represent extracellular FGFR inhibitor and selective FGFR inhibitors originated from natural products
